# A mapping review of Pacific Vascular Symposium 6 initiatives

**DOI:** 10.1016/j.jvsv.2023.101723

**Published:** 2023-12-20

**Authors:** Oscar Moreno, Kiran Kumar, Fedor Lurie, Marc A. Passman, Glen Jacobowitz, Faisal Aziz, Peter Henke, Thomas Wakefield, Andrea Obi

**Affiliations:** a Section of Vascular Surgery, Department of Surgery, University of Michigan, Ann Arbor, MI; b Jobst Vascular Institute of ProMedica, Toledo, OH; c UAB Vein Program and Clinic, Department of Surgery, The University of Alabama at Birmingham, Birmingham, AL; d Section of Vascular Surgery, Department of Surgery, New York University, New York, NY; e Division of Vascular Surgery, Department of Surgery, The Pennsylvania State University, Hershey, PA

**Keywords:** Chronic venous disease, Mapping review, Pacific Vascular Symposium

## Abstract

**Objective:**

The 2010 Pacific Vascular Symposium 6 (PVS6) brought venous disease content experts together with a goal of addressing critical issues collated together in the next decade with concrete plans to achieve these goals. This mapping review aims to provide a broader representation of how progress in critical issues of chronic venous disease has been made by extrapolating scientific publications related to the PVS6 initiatives.

**Methods:**

We performed a mapping review identifying original or systematic review/meta-analysis articles related to PVS 6 initiatives (aims) that addressed one of the following key objectives: scales to measure chronic venous disease, effectiveness of interventional deep venous thrombus removal, development of a deep venous valve, and biomarkers related to venous disease. Searches were undertaken in PubMed, Ovid Medline, Cochrane Library, Embase (Elsevier), CINAHL (EBSCO), and Scopus. We extracted descriptive information about the studies and predefined variables for each specific aim, showing what and where research exists on the aims included.

**Results:**

A total of 2138 articles were screened from 3379 retrieved articles from six electronic databases. We mapped 186 included articles, finding that the total number of publications significantly increased after the 2010 PVS6 meeting. Aim results were visually summarized. The largest body of data addressed catheter-based thrombus removal strategies for acute iliofemoral deep venous thrombosis. Primary research on artificial venous valves and venous biomarkers remained limited. No new post-thrombotic syndrome (PTS) score has been developed.

**Conclusions:**

This mapping review identified and characterized the available evidence and gaps in our knowledge of chronic venous disease that exist visually, which may guide where more significant investments for the future should be targeted.

The Pacific Vascular Symposia (PVS) (1993-2010) were established as forums for venous experts to address clinical priorities in venous research, implementation, and advocacy. Historically, the PVS symposia series have produce landmark venous interventions such as the Clinical (C), Etiological (E), Anatomical (A), and Pathophysiological (P) (CEAP) score, commonly use in clinical practice.^1^, ^2^, ^3^ The most recent PVS6 meeting was held in 2010 to identify measures to decrease the incidence of venous ulceration by 50% in 10 years.^4^ Globally invited clinician experts and representatives from health care management, the insurance industry, marketing industry research and development, and public policy experts met over 4 days in November 2009.^5^ Sixty participants were chosen based on a combination of expertise in venous disease, wound care, internal medicine, nursing, vascular technology, basic science, epidemiology, health care management, insurance industry, public policies, marketing, industry research and development, and government relationships.^6^ The aim was to arrive at a reasonable number of critical issues and devise a detailed action plan. The meeting was summarized in six major topic initiatives (Table I).^4^^,^^5^ No further PVS have occurred since 2010; thus, progress on these initiatives has yet to be measured.

**Table I:** Pacific Vascular Symposium 6 (*PVS6*) priorities and included aims

PVS6 priorities - the next steps	PVS6: a mapping review
1. Major program of awareness of venous ulcer and CVD, both professional and public	
2. Standardization of the diagnosis of CVD	Initiative 1: The need to develop a validated standard scale to measure CVD, improved over the Villalta scale.
3. Prevention of the PTS	Initiative 2: The need to determine if PMT is useful for both iliofemoral DVT and femoropopliteal DVT.
4. Treatment of C4 to 6 patients, including those with venous ulcers, including compression, correction, and surveillance guidelines	Initiative 3: The need to develop a functional venous valve and determine in which setting(s) such a venous valve would be useful.
5. Research in CVD	Initiative 4: The need to define biomarkers to help determine which patients with C4 disease will progress to C5/C6 disease.
6. Organizational goals in CVD.^4^	

*CVD*, Chronic venous disease; *DVT*, deep venous thrombosis; *PMT*, pharmacomechanical thrombolysis; *PTS*, post thrombotic syndrome.

A mapping review (MR) is a mechanism to systematically identify, describe, and characterize evidence in research in a particular area from available studies from predefined variables. Results are commonly summarized visually as bubble maps, multi-axes charts, and time-based trend graphics. It is the preferred methodology for investigating gaps in the literature.^7^, ^8^, ^9^ We performed a MR for four relevant research questions remaining from PVS6. This review aims to identify trends in evidence production of remaining gaps, determine major thematic directions of the published work after 2010 compared with before PVS6, and summarize these results visually.

## Methods

### Mapping review methodology

We aim to identify, describe, catalog, and summarize the available research on chronic venous disease (CVD) and evidence gaps regarding critical issues identified in the PVS6 meeting. This meeting addressed six topic initiatives, two related to program awareness and system organization (#1, #6). The remaining four were patient care-related (#2-#5) (Table I). From the six topic initiatives, we identified four questions that could be answered by a literature search involving patient care priorities. Consensus on these questions for the MR was obtained at the 2022 American Venous Forum research retreat. With this methodology, we extracted descriptive information about the studies and predefined variables for each specific aim, showing what and where research exists on the aims included. We included original, quantitative, qualitative, and mixed methods articles, qualitative evidence, and health policy/management information published in peer-reviewed journals within 10 years before PVS and up to 2022, available in English. We excluded articles that did not meet our research question of key CVD impact areas. We also excluded articles not available in English and narrative reviews.

### Information sources

We retrieved, reviewed, and filtered by standard literature search methods using the search algorithms described in the Supplementary Materials in the electronic databases: PubMed, Ovid Medline, Cochrane Library, Embase (Elsevier), CINAHL (EBSCO), and Scopus. The translation or conversion between the different electronic databases was done using Polyglot’s algorithm translation tool (https://sr-accelerator.com/#/polyglot).

### Selection process

A panel of experts (O.M., A.O., T.W., P.H.) selected articles with a minimum of two reviewers. The duplicated article exclusion, inclusion, and initial database construction was done using the Rayyan platform (https://www.rayyan.ai), sharing access accounts with the reviewers and the project manager. A blinded selection of articles was used. If the inclusion of an article was found to be in conflict between the two reviewers, a third reviewer adjudicated the decision.

### Extraction methodology

Included articles were first classified by their year of publication as pre- or post-PVS6. We extracted descriptive information (study design, year, and author’s country), predefined general variables (author’s country, world region [Americas, Europe, Asia, and Oceania], sample size, methodological methods, outcomes, CVD impact area, and other variables), and specific predefined variables for each aim (Table II).

**Table II:** Predefined variables extracted by aim from the Pacific Vascular Symposium 6 (PVS6)

Aim 1	Aim 2
Each scale implemented and combinations. (CEAP, Villalta, VCSS, AVVQ, VEINES QOL, SF-36, CIVIQ, EQ-5D, and other), reported uses of these scales (comparative, diagnosis, QoL relationships, and scale validation)	Type of study, location of the intervention (IVC/Iliac, iliofemoral, femoropopliteal), administered treatment [anticoagulation as medical management], surgical thrombectomy/hybrid, CDT, PMT/MT/PAT, PCDT, and USCDT. Also, total number of cases, the timing of intervention (acute, subacute, chronic), indication (DVT, PTS), the clinical scales implemented (CEAP, Villalta, VCSS/rVCSS, SF-36, VEINES-QoL-Sym, CIVIQ, other), and the use of adjunctive therapies (stenting, balloon angioplasty, IVC filter).

Aim 3	Aim 4
Study classification: preclinical (in vitro, in silico/CFD), structural analysis, or clinical (clinical trials), indication of the study (CVI, PTS), the main objective (valve lesion analysis, structural analysis, implantation analysis), a method for preoperative and follow-up evaluation (ultrasound/IVUS), the research outcome if reported, adjunctive treatments used (anticoagulation, IVC filters), clinical outcomes (patency, symptoms resolutions), complications if reported, and follow-up time in months.	Each biomarker reported, the indications, article type, if there was any follow-up (months), the clinical scales reported, outcomes if reported, and the primary use for the biomarkers (associations or prognosis), and biomarker categories: inflammation, coagulation and fibrinolysis, adhesion molecules, iron metabolism and oxidative stress, extracellular matrix, tissue remodeling, immune system, hormones, metabolism, growth factors, neurotransmitters, organ function, and tissue perfusion.

*AVVQ*, Aberdeen Varicose Vein Questionnaire; *CDT*, catheter-directed thrombolysis; *CEAP*, Clinical (C), Etiological (E), Anatomical (A), and Pathophysiological (P); *CFD*, computer fluid dynamics; *CIVIQ*, Chronic Venous Insufficiency Quality of Life Questionnaire; *CVI*, chronic venous insufficiency; *DVT*, deep vein thrombosis; *EQ-5D*, EuroQol 5D instrument; *IVC*, inferior vena cava; *IVUS*, intravascular ultrasound; *MT*, mechanical thrombectomy; *PAT*, percutaneous aspiration thrombectomy; *PCDT*, pharmacomechanical catheter-directed venous thrombolysis; *PMT*, pharmacomechanical thrombolysis; *PTS*, post thrombotic syndrome; *QoL*, quality of life; *SF-36*, 36-Item Short Form; *USCDT*, ultrasound-assisted catheter-directed thrombolysis; *VCSS*, Venous Clinical Severity Score; *VEINES QOL*, Venous Insufficiency Epidemiological and Economic Study - Quality of Life/Symptoms.

### Data management, statistical analysis, and the synthesis process

The selected articles were reviewed and codified in a Microsoft Excel database by aims. We summarized the preselected extracted variables from all aims using descriptive statistics in SPSS (v28, IBM Corp) and Prism (v9, GraphPad). Visual summaries were developed using Adobe Illustrator from the aggregate data extracted for each specific aim.

Differences in article interconnectedness was represented by mapping the citations among the included articles, or related to the PVS6 original publications, using Litmaps (https://www.litmaps.com/), a citation analysis platform. If other authors cite other articles in the same aim, a connecting line is shown between both articles. This relationship can be used to infer if the authors are considering society/expert recommendations such as the PVS6 meeting, by summarizing the citations between the studies.^10^ Finally, all the included biomarkers were imported into String DB (https://string-db.org/) to identify and summarize the associated protein networks related to CVD.

## Results

**Fig 1:**
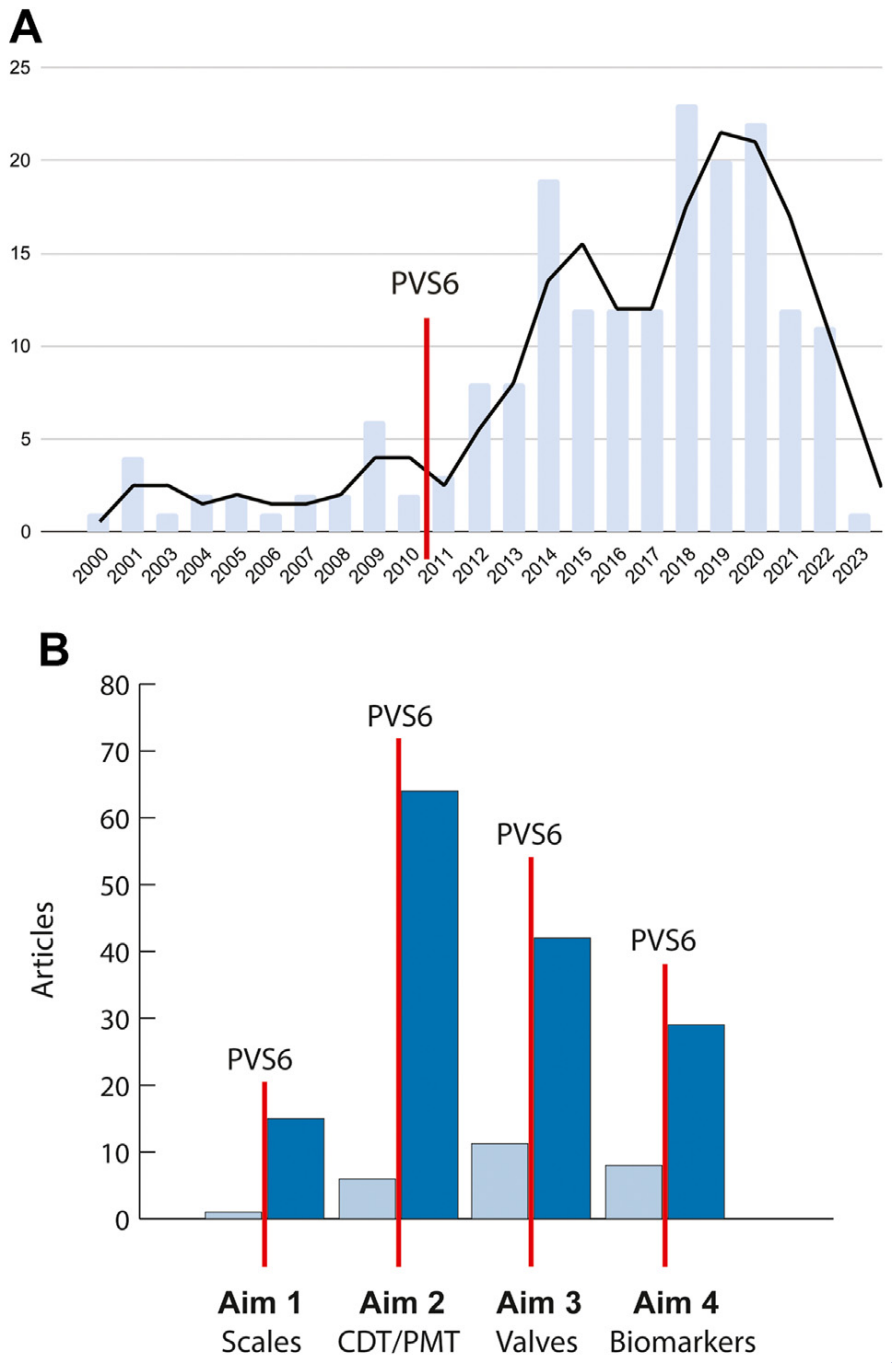
**A,** Number of publications by year before and after December 2010 (Pacific Vascular Symposium 6 [*PVS6*] publication date). **B,** Number of publications by year before and after December 2010 (PVS6 publication date) filtered by aim. *CDT*, Catheter-directed thrombolysis; *PMT*, pharmacomechanical thrombolysis.

**Supplementary Fig 1:**
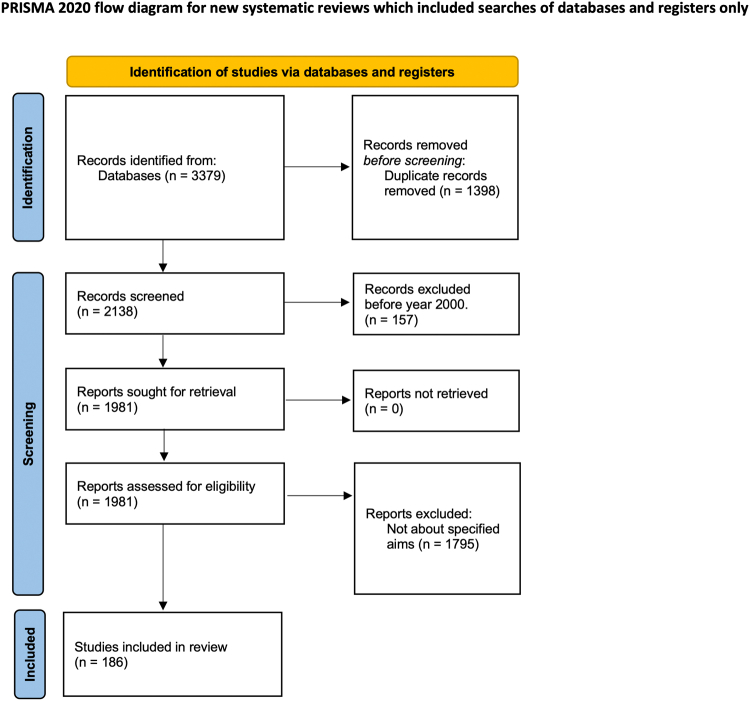
Flow diagram of the search methodology. *PRISMA*, Preferred Reporting Items for Systematic Reviews and Meta-Analyses.

**Supplementary Fig 2:**
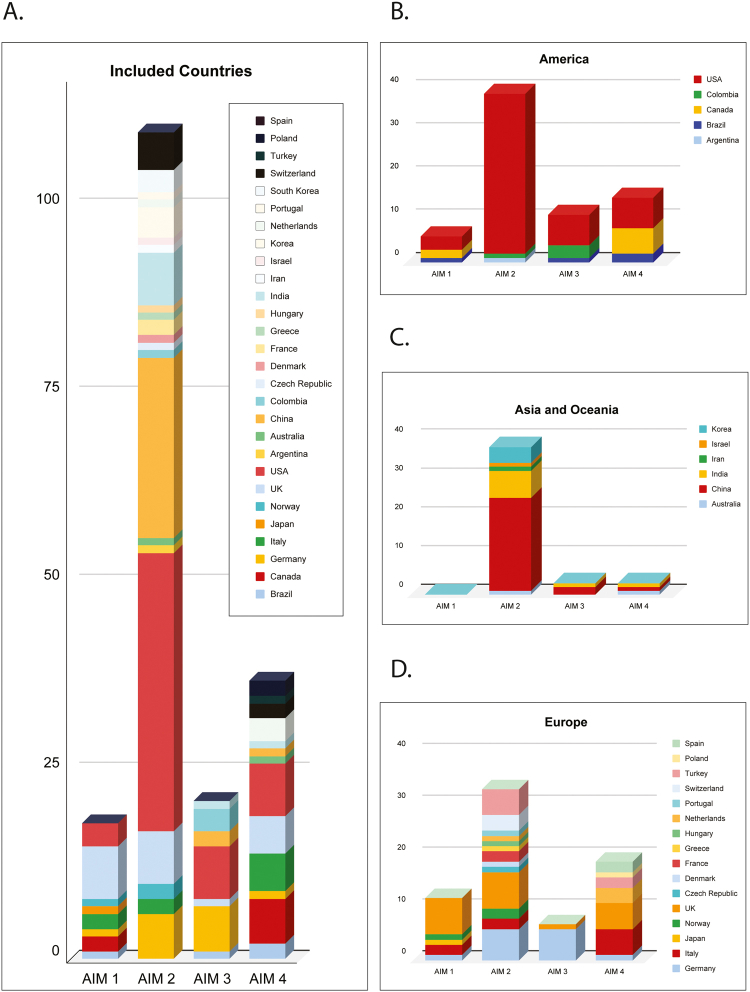
**A,** Author country origin of the included articles. **B,** Selective author distribution in America. **C,** Selective author distribution in Europe. **D,** Selective author distribution in Asia. *UK*, United Kingdom; *USA*, United States.

A total of 3379 articles were retrieved from six electronic databases. After removing 1398 duplicate articles, 18 were included in Aim 1, 110 in Aim 2, 21 in Aim 3, and 37 in Aim 4. The flow diagram of the search methodology is shown in Supplementary Fig 1. The total number of relevant publications demonstrated a 6.8-fold increase after December 2010 (PVS6 publication date) (Fig 1, *A*). The greatest increase in published manuscripts was related to thrombolysis/thrombectomy (11-fold) and scales of CVD (17-fold). The smallest fold increase was observed in studies about biomarkers (3.6-fold) and artificial venous valve articles (2.5-fold) (Fig 1, *B*). We found wide diversity in publication origins: the countries with the highest number of included publications were the United States (29%), China (14.5%), the United Kingdom (10.7%), and Germany (7.5%) (Supplementary Fig 2, *A-D*).

### Aim 1: the need to develop a validated standard scale to measure chronic venous disease improved over the Villalta scale

**Fig 2:**
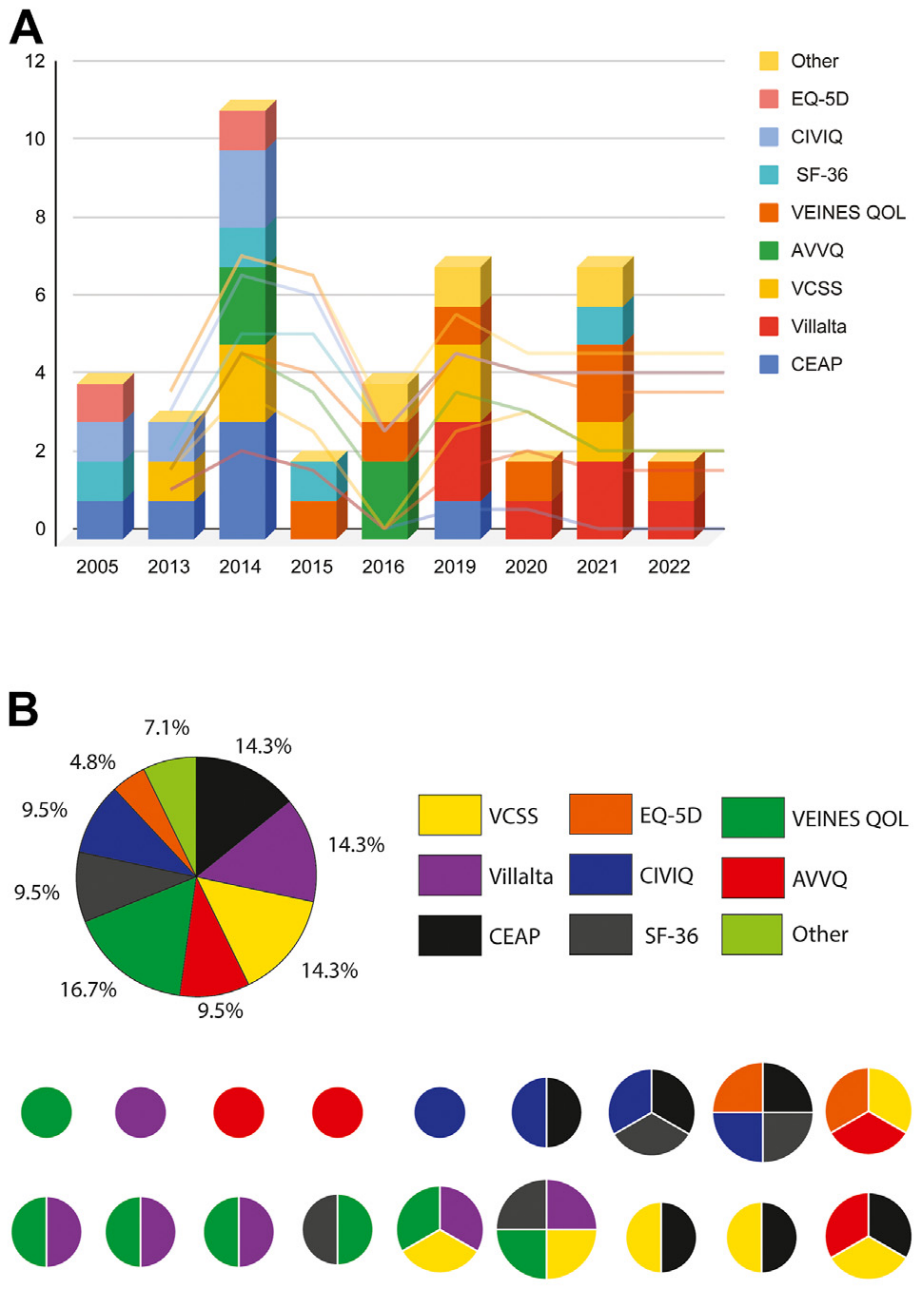
**A,** Overall use of clinical and quality of life (QoL) scales uses by year. **B,** Clinical and QoL overall scale use and scales combinations by the number of patients enrolled. *AVVQ*, Aberdeen Varicose Vein Questionnaire; *CEAP*, Clinical (C), Etiological (E), Anatomical (A), and Pathophysiological (P); *CIVIQ*, Chronic Venous Insufficiency Quality of Life Questionnaire; *EQ-5D*, Euro QoL-5D instrument; *SF-36*, 36-item Short Form Survey; *VCSS*, Venous Clinical Severity Score; *VEINES QOL*, Venous Insufficiency Epidemiological and Economic Study - Quality of Life/Symptoms.

We analyzed studies reporting on venous disease severity scales and quality of life (QoL) measures. We found no new tools to measure PTS during this time. Villalta, CEAP, and Venous Clinical Severity Score (VCSS) were studied at equal frequency (Fig 2). However, there was a shift in publications utilizing QoL scores. Venous disease and QoL research arose predominantly from Europe, whereas valvular research was primarily performed in the Americas (Supplementary Fig 2, *A-D*). From 2006 to 2016, we found that the Chronic Venous Insufficiency Quality of Life Questionnaire (CIVIQ), 36-Item Short Form Health Survey (SF-36), and Aberdeen Varicose Vein Questionnaire (AVVQ) were the most frequently studied scales compared with the period from 2019 to 2022, in which the primary scale studied was the Venous Insufficiency Epidemiological and Economic Study Questionnaire (VEINES-QoL). We also analyzed the most frequent combination of scales used and found Villalta and VEINES-QoL to be the most common combination. We also found that the most common studies for these scales were exploring QoL relationships with other clinical variables in 50%, comparing scales in 28%, scale validation in 24%, and their use in diagnosis in 8%.

### Aim 2: the need to determine if pharmacomechanical thrombolysis is useful for both iliofemoral and femoropopliteal deep venous thrombosis

**Fig 3:**
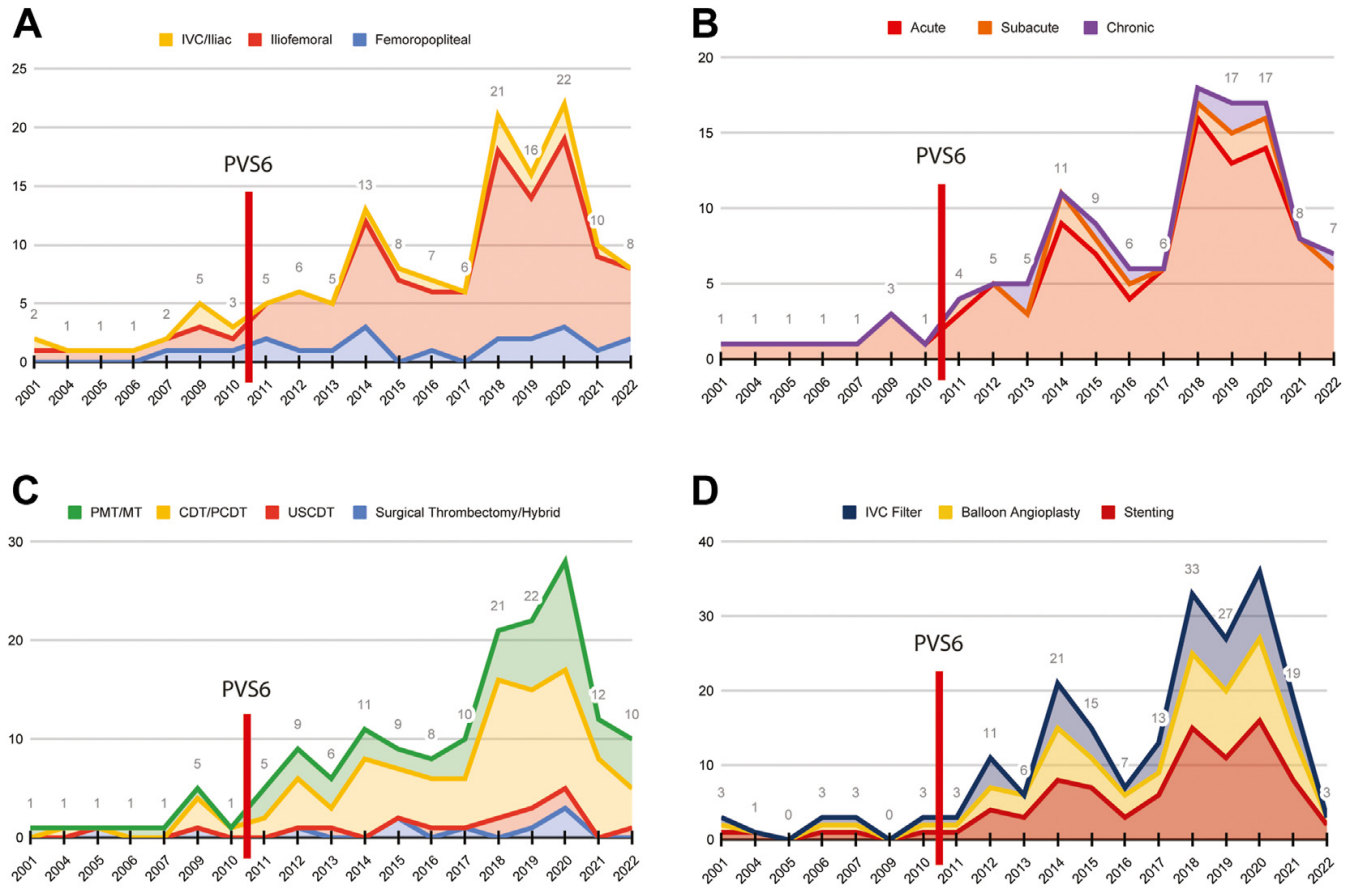
**A,** Location of deep venous thrombosis (DVT) for intervention, including iliofemoral, femoropopliteal, and inferior vena cava (*IVC*) and iliac segment before and after the Pacific Vascular Symposium 6 (*PVS6*) publication. **B,** Reported chronicity in DVT treatment, including acute, subacute, and chronic settings before and after the PVS6 publication. **C,** Use of catheter-directed thrombolysis (*CDT*)/ pharmacomechanical catheter-directed venous thrombolysis (*PCDT*), followed by pharmacomechanical thrombolysis (*PMT*)/ mechanical thrombectomy (*MT*), ultrasound-assisted catheter-directed thrombolysis (*USCDT*), and surgical thrombectomy and hybrid techniques for DVT treatment before and after the PVS6 publication. **D,** Use of adjunct procedures for DVT treatment before and after the PVS6 publication.

We found that 22% of studies involved multi-segment disease. Among all studies, including those involving multiple segments, the most common location for intervention was iliofemoral (90.9%), followed by femoropopliteal (18.1%), inferior vena cava (IVC), and iliac segments (13.6%) (Fig 3, *A*). Most publications reported on interventions for acute deep venous thrombosis (DVT) (89%). Approximately 9.5% of studies included DVT of varying chronicity. A minority of articles included patients with subacute (9%) and chronic DVT (8.1%) (Fig 3, *B*). We found an increase in overall procedural publications after the PVS6 publication. Approximately 51% of papers cited multiple indications for interventions: primarily DVT in 80.9% and PTS in 63.6%. Among procedure types, catheter-directed thrombosis (CDT)/ pharmacomechanical catheter-directed venous thrombolysis (PCDT) was the most frequent procedure performed (86%), followed by pharmacomechanical thrombolysis (PMT)/mechanical thrombectomy (MT) (48%). Ultrasound-facilitated catheter-directed thrombolysis (USCDT) (8.1%), surgical thrombectomy, and hybrid techniques were the most minor used interventions (8.1%) (Fig 3, *C*). The most reported adjuvant procedure was venous stenting (77.2%), followed by balloon angioplasty (56.3%) and the placement of an IVC filter (45.4%) (Fig 3, *D*). The most reported clinical scale related to interventions was Villalta (70%), followed by CEAP (16.3%), VSCSS/rVCSS (14.5%), VEINES-QoL/Sym (8.1%), SF-36 (2.7%), CIVIQ (3.6%), and other reported scales (5.4%).

### Aim 3: the need to develop a functional venous valve and determine which setting(s) such a venous valve would be useful

We determined that the settings for functional artificial venous valves (AVVs) implanted were in chronic venous insufficiency (CVI) (14/16 trials) and post-thrombotic syndrome (PTS) (2 of 16 trials). An increase in studies from two to seven post-PVS6 addressing the structure of valves was observed. Preclinical studies accounted for 28% of the publications, including computational fluid dynamics and other computer-aided tools. We found standardized pre- and postoperative evaluation and follow-up after the implantation of AVVs reported in 72% of the included articles. A shift in imaging from phlebography to duplex was noted in the post-PVS6 era.

**Fig 4:**
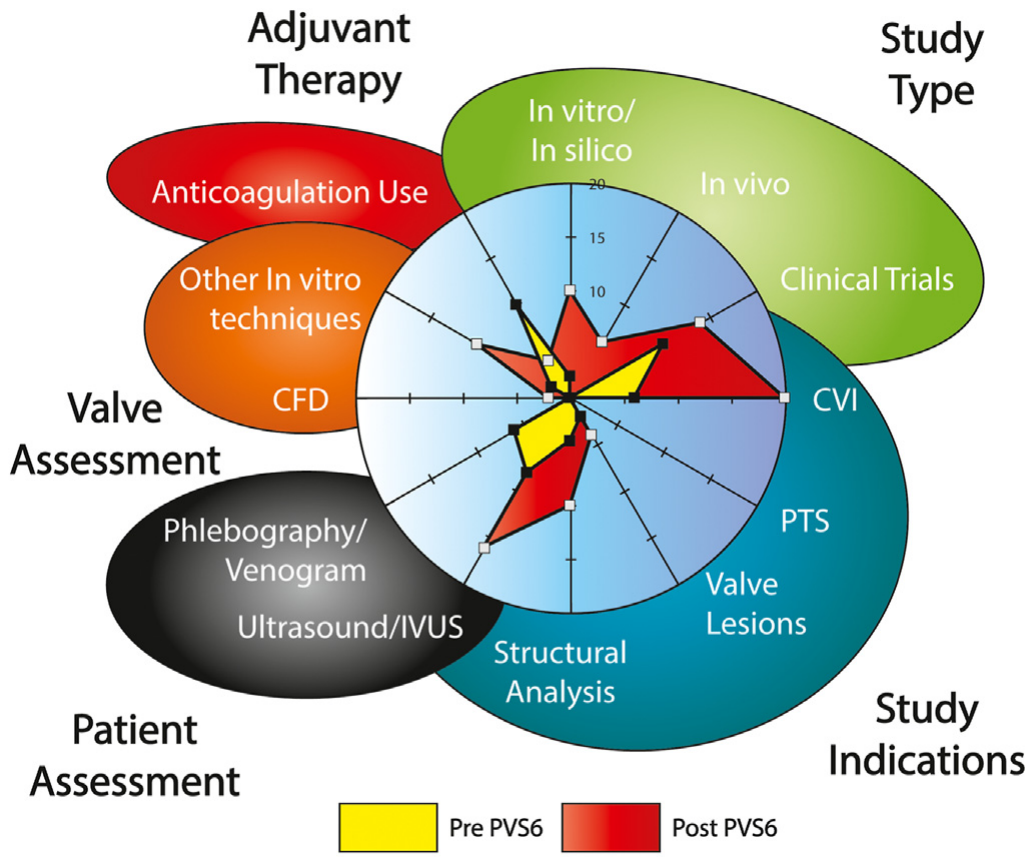
Artificial venous valve (AVV) study type, indication, preclinical and clinical valve assessment, and anticoagulation reported use. *CFD*, Computer fluid dymanics; *CVI*, chronic venous insufficiency; *IVUS*, intravascular ultrasound; *PTS*, post thrombotic syndrome.

Ultrasound/intravascular ultrasound (IVUS) use was noted in 56% and was more common after PVS6, with phlebography/venogram at 16%, primarily in the pre-PVS6 era. The use of adjunctive treatments (anticoagulation, IVC filter, other) was reported in 36% of the articles. Clinical outcomes were reported in 64%, and follow-up was reported in 52% of the studies (with a mean of 8.9 ± 16.7 months.) These findings are shown in Fig 4, demonstrating the shift in publication topic areas before and after PVS6 (pre-PVS6 in yellow, post-PVS6 in red).

### Aim 4: the need to define biomarkers to help determine which patients with C4 disease will progress to C5/C6 disease

**Fig 5:**
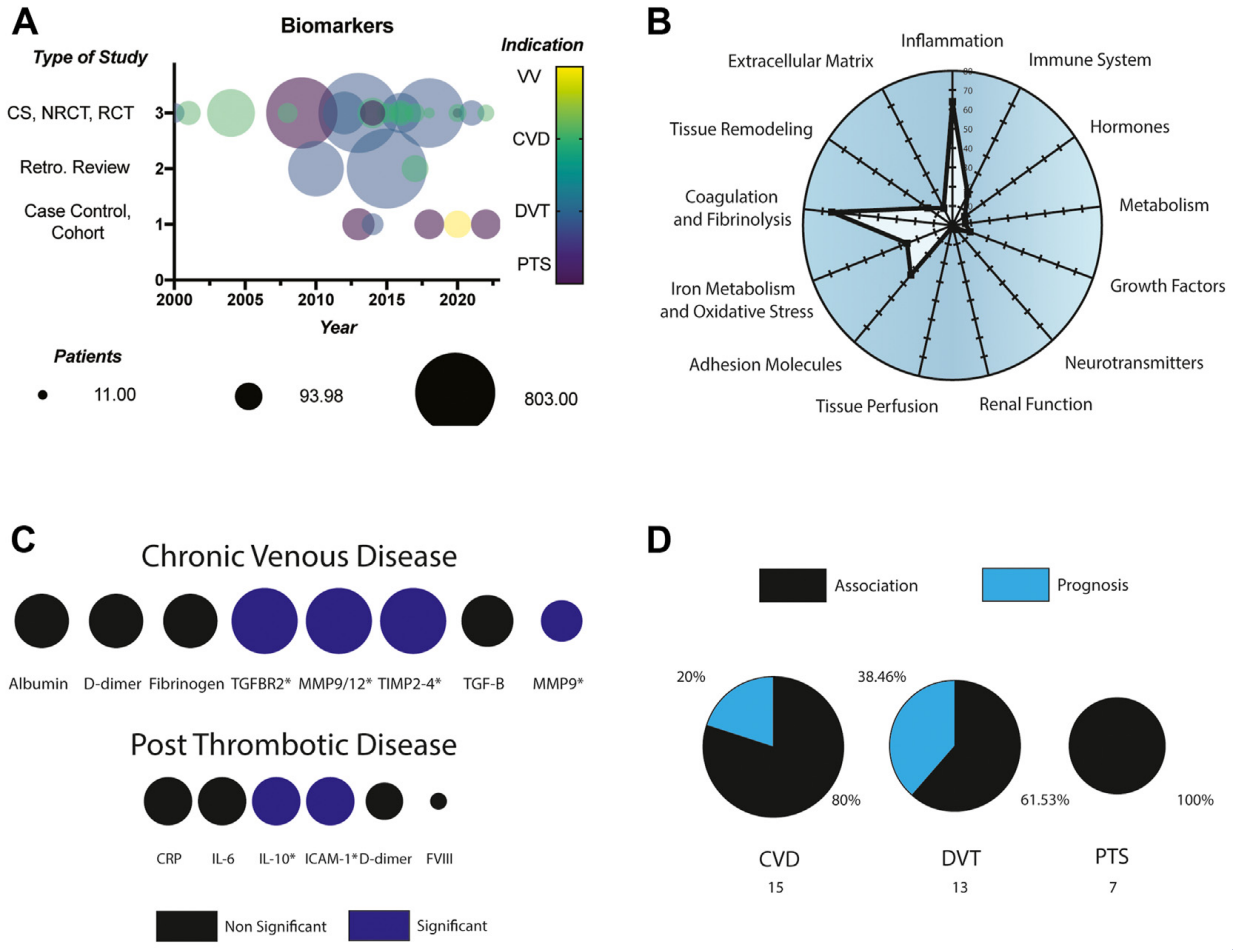
**A,** Study types, indication, and the number of patients enrolled in chronic venous disease (*CVD*) research. **B,** Subgroup classification of the reported biomarkers in CVD research. **C,** Most common biomarkers associated with CVD and post thrombotic syndrome (*PTS*). **D,** Reported biomarker association or prognosis findings in patients with CVD, deep venous thrombosis (*DVT*), PTS. *CS*, Clinical study; *NRCT*, non-randomized observational study; *RCT*, randomized clinical trial; *VV*, varicose veins.

Following the PVS6, we found that the most substantial number of studies were devoted to biomarker research related to CVD, followed by DVT, varicose veins (VVs), and PTS. Many systematic reviews and meta-analyses were dedicated to DVT. However, the primary literature was restricted to only eight studies (21%). PTS-related biomarker research was primarily conducted as case-control/cohort studies (Fig 5, *A*). Many retrospective reviews were found to be related to DVT. Most of the case-control and cohort studies with fewer patients were primarily associated with PTS, followed by DVT and varicose veins. Most biomarker studies involved molecules related to inflammation, coagulation and fibrinolysis, adhesion molecules, iron metabolism, and oxidative stress (Fig 5, *B*). All the included biomarkers are reported in Table III.

**Table III:** The total included biomarkers associated with venous disease

Inflammation	64	Immune system	18	Metabolic markers:	7	Adhesion molecules	34	Iron metabolism and oxidative stress	26	Coagulation and fibrinolysis	65	Tissue remodeling	16
CRP/hs-CRP	12	CD4+	1	Lipoprotein A	1	ICAM-1	6	MCP-1	5	LAC	2	MMP-1	1
IL-6	16	CD8+	1	Folate	1	VCAM-1	2	PAI-1	2	Antiphospholipid antibodies	2	MMP-8	1
IL-10	4	CD19+	1	Vitamin B12	1	LFA-1	1	FPN1-8CG	1	FXIII V34 L	4	MMP-9	5
TNF-α	2	Mastocytes	1	MYOD signature genes	1	Mac-1	1	HFE C282Y & H63D	1	FVIII	6	TIMP-1	1
CCL11/Eotaxin	1	Neutrophil count	1	Glucose	1	p150,95	1	HIF-1α/HIF-2α	2	ATIII	4	TIMP-2	1
IFNa2	1	Neutrophil-lymphocyte ratio	1	mRNA PI3K/Akt/mTOR	1	CD18	1	Iron	2	PS	4	MMP-2	2
IFNg	1	MPV	1	Angiotensin-converting enzyme activity	1	VLA-4	1	Malonyldialdehyde	1	PC	6	MMP-3	1
MIP1a	1	WBC	1	Growth factors	10	L-selectin	2	Total antioxidant capacity	1	FVL	7	MMP-7	1
MIP-1b	1	Platelets	3	NO	1	E-selectin	1	Uric acid	1	PTM	1	MMP12-82AG	1
IL-12P4	1	Leukocytes	1	VEGF	2	sICAM-1	3	% transferrin iron binding capacity	1	ACL	1	Haemoglobin	3
IL-12P7	1	Platelet activation status	1	FGF	1	sVCAM-1	2	Ferritin	1	Prothrombin	7	TIMP-3/4	1
IL-13	1	Granulocyte pseudopod formation	1	G-CSF	1	sE-selectin	1	Transferrin	1	D-dimer	7	Extracellular matrix	10
IL-15	1	Hydrogen peroxide production	1	CM-CSF	2	P-selectin	1	Lactoferrin	1	Cardiolipin antibodies IgGtype	1	Elastin	1
IL-17A	1	Nitroblue tetrazolium reduction	1	PDGF-BB	1	CD11a (b2-integrin a-chain)	1	Thrombomodulin	1	ADAMTS13	1	Tropoelastin	1
IL-1RA	1	Leukocyte flow properties, WBC and PMN characteristics	1	TGFBR	1	CD11b (b2-integrin a-chain)	1	Hemochromatosis	1	APTT	1	Fibrilin-1	1
IL-b	1	Monocyte proliferation	1	TGF-b	1	CD15 (Lewis' antigen X)	1	Collagen	2	TT	1	Fibulin 4	1
IL-2	1	Hormones	8	Neurotransmitters	3	CD31 (platelet-endothelial adhesion molecule-1)	1	CASZ1	1	TM	1	Fibulin 5	1
IL-4	1	Androstendione	1	Glutamate	1	CD54 (intercellular adhesion molecule-1)	1	Homocysteine	1	Fibrinogen	3	LOX	1
IL-5	2	Dehydroepiandrostendion	1	Taurine	1	CD49 d (a-chain of very late antigens-4)	1	Renal function	2	TAT	1	LOXL1	1
IL-7	1	Estradiol	2	Myo-inositol	1	CD62 L (L-selectin)	1	NGAL (kidney)	1	Elastase	1		
IL-8	5	Testosterone	2			INT reduction	1	Peak oxygen consumption (PVO)	1	Lysozyme	1		
IP-10	1	Insulin	1			Superoxide	1	Respiratory function	1	Myeloperoxidase	2		
vWF	2	Sex hormone binding globulin	1			RBC CD35 expression	1			Beta-D-glucuronidase	1		
Intratrombocyte cytologic calcium concentration	1					RBC Fy6 expression	1						
ADP-induced trombocyte aggregation	1												
APC resistance	2												

*ACL*, anticardiolipin antibody; *ADP*, adenosine diphosphate; *APC*, activated protein C; *ATIII*, antithrombin III, *CASZ1*, castor zinc finger 1; *CCL*, CC motif chemokine ligand; *CM-CSF*, granulocyte-macrophage colony-stimulating factor; *CRP*, C reactive protein; *FGF*, fibroblast growth factor; *FPN1-8CG*, ferroportin-1; *FVIII/IX/XI*, Factor VIII/IX/XI; *FVL*, Factor V Leiden; *G-CSF*, granulocyte colony stimulating factor; *HFE*, hemochromatosis gene; *HIF-1/2α*, hypoxia-inducible factor 1/2-alpha; *hs*, high sensitivity; *ICAM-1*, intercellular adhesion molecule 1; *INT*, 3-(4-iodophenyl)-2-(4-nitrophenyl)-5-phenyltetrazolium chloride; *IP-10*, interferon γ-induced protein 10; *L*, interleukin; *LAC*, lupus anticoagulant; *LFA*, Lymphocyte function-associated antigen; *LOX*, lysyl oxidase; *LOXL1*, lysyl oxidase like 1; *Mac-1*, macrophage-1 antigen; *MCP-1/CCL2*, monocyte chemoattractant protein-1; *MIP*, macrophage inflammatory protein; *MMP*, matrix metalloproteinase; *MPV*, mean platelet volume; *mRNA*, messenger RNA; *MYOD*, myogenic regulatory protein; *NGAL*, neutrophil gelatinase-associated lipocalin; *NO*, nitric oxide; *PAI-1*, plasminogen activator inhibitor 1; *PC*, protein C; *PDGF-BB*, platelet-derived growth factor; *PS*, protein S; *PTM*, prothrombin mutation; *PVO*, peak oxygen consumption; *RBC*, red blood cell; *sICAM-1*, soluble intercellular adhesion molecule-1; *sVCAM-1*, soluble vascular cell adhesion molecule-1; *TAT*, thrombin anti thrombin complexes; *TE*, tropoelastin; *TGF-b*, transforming growth factor beta; *TGFβR2*, transforming growth factor beta receptor 2; *TIMP*, tissue inhibitors of metalloproteinases;*TM*, thrombomodulin; *TNF*, tissue necrosis factor; *IFN*, interferon; *TT*, thrombin time; *VCAM-1*, vascular cell adhesion molecule 1; *VEGF*, vascular endothelial growth factor; *VLA-4(α4β1 integrin)*, very late antigen-4; *vWF*, Von Willebrand factor; *WBC*, white blood cells.

Biomarkers were primarily studied as associations with the disease state, but in a minority of studies, they were evaluated as predictive tools to predict progression from less severe to C5/6 disease (Fig 5, *C*). Significant associations were reported for CVD with TGFβR2, MMP9/12, TIMP 1-4, and for PTS with IL-10 and ICAM-1. Other non-significant associations that were commonly measured included albumin, d-dimer, fibrinogen, TGFb for CVD, and CRP, IL-6, D-dimer, and FVIII for PTS. We also extracted from the included biomarkers if they were involved in association or prognosis findings for CVD. We found that 80% of the reported biomarkers were described as associations and only 20% as prognostic factors. In the case of DVT, 62% of the biomarkers were described as associations and 38% as prognostic factors, and in the case of PTS, all were described as associations. (Fig 5, *D*).

**Supplementary Fig 3:**
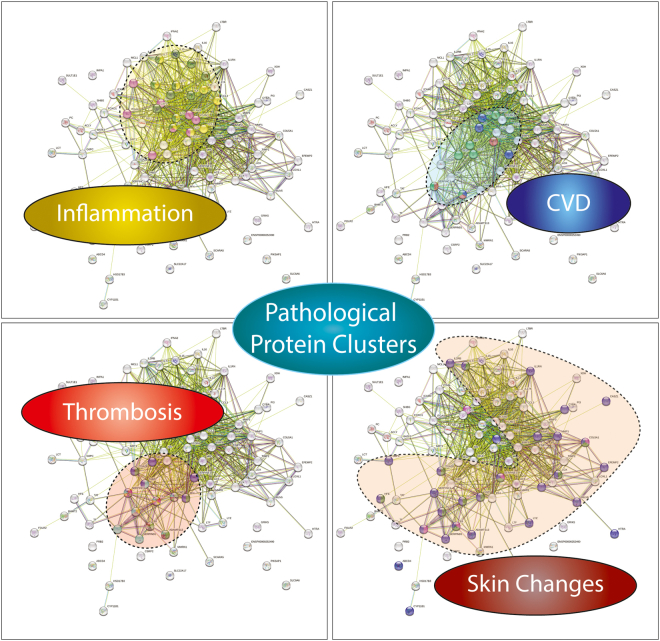
Main pathological protein clusters associated with inflammation, chronic venous disease (*CVD*), thrombosis, and skin changes.

Finally, we analyzed the relationship between the included protein biomarkers (protein-protein interaction network and functional enrichment analysis) using bioinformatic tools. This analysis revealed common pathological protein clusters associated with inflammation, CVD, thrombosis, and skin changes to be interconnected. (Supplementary Fig 3).

### How are these aims connected to the PVS6?

**Supplementary Fig 4:**
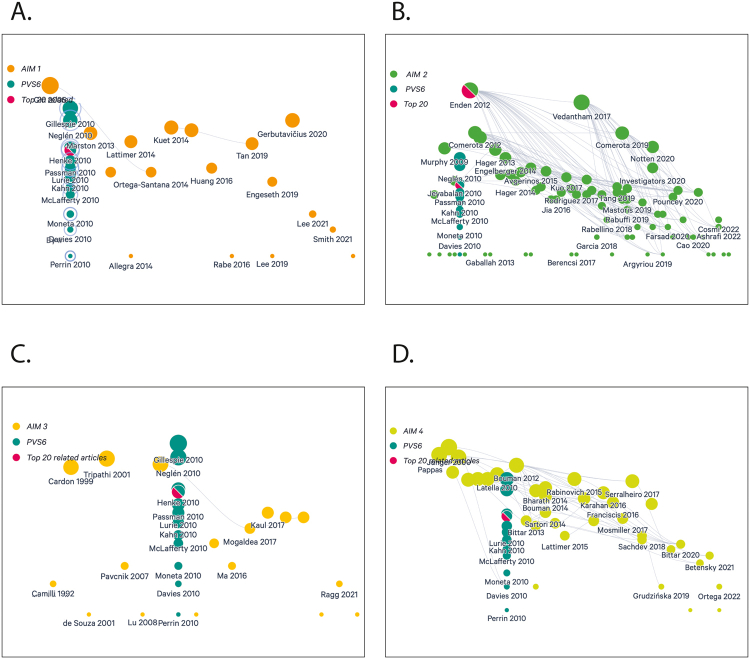
Visual summaries generated showing the aggregate data extracted for each specific aim. We show the differences in the degrees of connectedness as an association parameter on our predetermined areas (aims) summarizing the relationship between the studies by direct citation. **A,** Visualization for included articles in aim 1 by direct citation of the Pacific Vascular Symposium 6 (*PVS6*) articles and in between them. **B,** Visualization for included articles in aim 2. **C,** Visualization for included articles in aim 3. **D,** Visualization for included articles in aim 4.

The presented summary of the progress of PVS6 priorities is variable. We applied a bioinformatics reference tool to analyze how the PVS6 principal statements by Henke in 2010^4^^,^^5^ (shown in dark green) and other PVS6 published articles^6^^,^^11^, ^12^, ^13^, ^14^, ^15^, ^16^, ^17^, ^18^, ^19^, ^20^, ^21^, ^22^, ^23^, ^24^, ^25^ relate to the included articles by aim. We created four sets of linked data or knowledge graphs, which are visual techniques that show the relationships between the articles in terms of citations by year from all the included articles. We found that the visualizations for Aim 1 and 3 were not as connected as those for Aim 2 and 4, which show a richer interconnected environment (Supplementary Fig 4, *A-D*).

## Discussion

The PVS symposia series, particularly PVS6, has had a significant impact on advancing the clinical care of patients with venous disease. Historically, the PVS symposia was the origin of the CEAP score commonly used in clinical practice today. From the six major critical issues identified during PVS6 that were translated into initiatives in 2010, progress has been made in the four initiatives. This MR summarized the identified available evidence and gaps in Table IV. The total number of relevant publications has increased after PVS6, albeit unevenly among these topic areas. A large number of publications address catheter-based thrombus removal strategies for acute iliofemoral DVT but less so for chronic and more distal DVT. Despite a well-established need, research on venous valves remains limited, encompassing many preclinical trials. Primary biomarker studies are less commonly performed than interventional trials, and no new PTS score has been developed.^26^, ^27^, ^28^

**Table IV:** Identified trends in evidence production, remaining gaps, and to major future directions by aim

PVS6	Evidence summary	Evidence gaps	Interconnection
Aim 1	• Villalta, CEAP, and VCSS commonly used. • Rise in VEINES-QoL usage occurred post-PVS6. • Limited development of new scores or validation studies of existing scores.	• **How can the diagnostic accuracy of the current PTS scores be improved upon?**	• Low
Aim 2	• Catheter-based thrombus removal strategies for acute iliofemoral DVT commonly studied (11× increase post PVS6): iliofemoral >femoropopliteal. • Adjunct therapies commonly reported: venous stenting, balloon venoplasty, and IVC filter use.	• What is the utility of thrombus debulking strategies in chronic or distal DVT and PTS? • What are the optimal indications for specific adjuvant therapies in context of thrombus removal?	• High
Aim 3	• 72% of trials preclinical; clinical trials primarily included new prototypes implanted with open surgery; outcomes reported in 64% of trials. • 56% of trials utilized ultrasound/IVUS perioperatively; increased after PVS6. • Adjunctive treatments (eg, anticoagulation, IVC filter) reported in 36% of the articles.	• What is the optimal biomaterial choice, valvular design and delivery system for AVVs? • Do artificial valves reverse or limit sequalae from PTS or severe CVI? • What is the optimal anticoagulation and follow up following valve implantation?	• Low
Aim 4	• Biomarkers were primarily studied as associations with the disease state rather than prognostic indicator. • Significant associations were reported for CVD with TGFβR2, MMP9/12, TIMP 1-4, and for PTS with IL-10 and ICAM-1. • Other commonly biomarkers included albumin, D-dimer, fibrinogen, TGFb for CVD, and CRP, IL-6, D-dimer, and FVIII for PTS.	• **What biomarker or combination thereof predicts development of advanced chronic venous disease?** • **What biomarkers predict development of PTS?**	• High

*AVVQ*, Aberdeen Varicose Vein Questionnaire; *CEAP*, Clinical (C), Etiological (E), Anatomical (A), and Pathophysiological (P); *CIVIQ*, Chronic Venous Insufficiency Quality of Life Questionnaire; *CVD*, chronic venous disease; *CVI*, chronic venous insufficiency; *DVT*, deep vein thrombosis; *EQ-5D*, EuroQoL 5D instrument; *ICAM-1*, intercellular adhesion molecule 1; *IL-10*, interleukin-10; *IVC*, inferior vena cava; *IVUS*, intravascular ultrasound; *MMP*, matrix metalloproteinase; *PTS*, post-thrombotic syndrome; *SF-36*, 36-Item Short Form; *TGFβR2*, transforming growth factor beta receptor 2; *TIMP*, tissue inhibitors of metalloproteinases; *VCSS*, Venous Clinical Severity Score; *VEINES QOL*, Venous Insufficiency Epidemiological and Economic Study - Quality of Life/Symptoms.

Bold-text boxes denote evidence gaps identified in the original PVS6 document.

In this review, we found no newly developed chronic venous insufficiency score. We found Villalta, CEAP, and VCSS use constant in the pre and post-PVS6 cohorts. However, a shift in the study of QoL tools from CIVIQ, SF/36, and AVVQ to VEINES-QoL occurred in the post-PVS6 era. Few studies (n = 4) were dedicated to validating or testing scales already in clinical use. Although rigorous assessment of PTS and CVD severity is essential to accurate interpretation of clinical trials, rigorous creation of scoring systems and subsequent validation studies can be costly. It may offer no monetary incentive to the funding agency. We also found low interconnectedness based on citation mapping, suggesting fewer active investigator interactions. Development of such scores may be undervalued by stakeholders interested in better understanding the pathophysiology of the disease or establishing new treatments as tangentially related.

In contrast, we found an exponential increase of newly published data since PVS6 regarding CDT/PCDT interventions for treating DVT. Nearly all studies (∼81%) evaluated the utility of these techniques in acute DVT. Concordantly, fewer studies were specifically aimed at studying PTS (51%). This data might suggest a relationship with differences in the market landscape and regulatory framework, especially with stents that were approved after 2010. Different from stents, multiple percutaneous mechanical thrombectomy devices were available before 2000, making it difficult to establish the impact of those specific device approvals in our results.^29^, ^30^, ^31^ However, we did not find any United States Food and Drug Administration-approved device that attempts to resolve the limitations of crossing and debulking chronic thrombi.^32^ The current knowledge gap in understanding the role of interventional therapies in chronic DVT and PTS is currently being addressed by the ongoing CTRACT trial (NCT03250247).^33^ Although not explicitly addressed in the search criteria, the role of adjuvant therapies (such as venous stenting, angioplasty, IVUS, etc) in acute and chronic DVT likely remains an area deserving further research. The increase in endovascular interventions and adjuvant procedures leads to the need to develop better monitoring tools, databases, and integrative management of these patients in the long term, as well as the recognition of specific training needed in venous endovascular training.^32^^,^^34^

In aim 3, we evaluated progress on functional AVV development. In contrast to the diverse and substantial number of clinical trials for thrombectomy devices, a minority of published articles (36%) involving AVVs were preclinical studies. Clinical trials largely included novel prototypes implanted with open surgery, making up only a few clinical articles published with a limited number of patients (28 on average). Several limitations that need to be addressed include the implantation methods, the planning of treatment of prosthesis complications (thrombosis, infection, displacement, rupture), better biomaterials, the need for other imaging methods, and the length of the follow-up time after implantation. The relative complexity of engineering required to build a long-term functional venous valve compared with acute thrombectomy devices may be one reason for the relatively slow progress in this area. We also found that there was less inter-relatedness in terms of publication citation among this cohort of selected articles, indicating that perhaps funding sources and investigators are operating in geographic or specialty silos.

Similarly, we found fewer publications in biomarker evaluation. Despite the lack of a sensitive and specific biomarker to predict the development of advanced CVD, we found few primary studies before or following the PVS6 that attempted to answer this question. A relatively small number of studies (n = 12; 37%) addressed PTS-related biomarkers as case-control/cohort studies. Several categories were associated with the disease state, but a minority of studies were evaluated as prognostic tools to predict progression from less severe to C5/6 disease. We hypothesize that biomarker data may be limited for several practical reasons. First, projects requiring longitudinal study of patients over months to years can be expensive and challenging to fund. Second, improvements in endovenous techniques to allow for in-office treatment with reduced morbidity perhaps have dampened enthusiasm for a biomarker predictive of conversion to advanced CVD. Third, the widespread availability of duplex ultrasonography and safer anticoagulants may have limited the need for an early DVT blood biomarker.

### Limitations

This study was designed to summarize information regarding the number and types of articles published concerning the PVS6 topics. Specific conclusions regarding the specific content of the articles are not available. Furthermore, two of the six objectives from the conference involved public awareness campaigns and organizational goals in CVD. Analysis of progress in these areas was not possible with this research methodology. Finally, the selection of these articles was restricted to those available in English. If there were many manuscripts from research groups that did not routinely publish in English, we could have overlooked important advances since 2010.

Regarding the MR methodology, as with many other systematic review methodologies, MR can incur publication bias, not including unpublished studies with non-favorable or negative results. Also, the quality of the evidence was not directly assessed with specific tools regarding the diverse types of articles included. Finally, the reported use of any extracted variables is limited to English only in the mentioned databases from 2000 to 2022. Additional studies might not be included from different regions of the world that could contribute to the selected aims.

## Conclusion

Each of the key questions presented in the four initiatives was associated with published studies following the PVS6. The PVS6 articles included in a special supplement of *The Journal of Vascular Surgery* have been cited >130 times, suggesting that these documents can provide meaningful context and framework for researchers publishing in the selected field. We noted significant variation in publishing trends among the four initiatives, suggesting that variables such as availability of industry and governmental funding, time, and investigator expertise may be outsized in initiating and completing studies beyond the clinical need for such studies. The role of the American Venous Forum and other surgical societies may help “right the ship” by identifying, promoting, and funding areas of need, particularly those that may lack obvious economic incentives to industry partners. Furthermore, the lack of completion of another PVS highlights the need for follow-up planning in societal campaigns, particularly about answering to the ultimate consumers—the patients. We propose that further societal initiatives or other methods of evidence gap mapping include planned reassessments to identify underserved patient populations and underfunded areas of clinical need. Furthermore, surveys of the impacted populations including both patients and investigators should be considered, for continuous improvement.

## Author Contributions

Conception and design: FL, MP, GJ, FA, PH, TW, AO

Analysis and interpretation: OM, KK, PH, TW, AO

Data collection: OM, KK, PH, TW, AO

Writing the article: OM, KK, TW, AO

Critical revision of the article: OM, KK, FL, MP, GJ, FA, PH, TW, AO

Final approval of the article: OM, KK, FL, MP, GJ, FA, PH, TW, AO

Statistical analysis: OM, TW, AO

Obtained funding: Not applicable

Overall responsibility: AO

## Disclosures

None.
