# Supplementary material for: A mapping review of Pacific Vascular Symposium 6 initiatives
Source: J Vasc Surg Venous Lymphat Disord. 2023 Dec 20;12(4):101723. doi: 10.1016/j.jvsv.2023.101723 (PMC11523466; doi:10.1016/j.jvsv.2023.101723)
Supplement: Supplemental Materials [file mmc1.docx]

**SUPPLEMENTAL MATERIALS**

**Search Strategy and Results 2010-2022**

| **KEY AREA** | **Aim 1** | **Aim 2** | **Aim 3** | **Aim 4** |
| --- | --- | --- | --- | --- |
| PUBMED | 121 | 264 | 35 | 415 |
| OVID MEDLINE | 109 | 267 | 63 | 297 |
| COCHRANE LIBRARY | 92 | 18 | 3866 | 95 |
| EMBASE (ELSEVIER) | 237 | 780 | 91 | 92 |
| CINAHL (EBSCO) | 20 | 60 | 17 | 75 |
| SCOPUS | 257 | 578 | 34 | 9010 |
| **TOTAL** | **579** | **1389** | **411** | **974** |
| Duplicates | 409 | 596 | 32 | 361 |
| For selection | **170** | **793** | **379** | **613** |
| **INCLUDED** | 18 | 110 | 21 | 37 |

1. ***Standardize diagnosis of CVD:*** *Need a validated standard scale to measure CVI, improved over the Villalta scale.

| **Database** | **Date** | **Search Algorithm** |
| --- | --- | --- |
| PUBMED= 121  1997-2022 | 02/09/22 | ((“Villalta”[tiab] OR “Ginsberg”[tiab] OR “Brandjes”[tiab] OR “Widmer”[tiab] OR“Marder” OR “VEINES-QOL”[tiab] OR VAS[tiab] OR VCSS[tiab] OR “Chronic Venous Insufficiency Questionnaire”[tiab] OR CIVIQ[tiab] OR ”Aberdeen Varicose Vein Questionnaire”[tiab] OR AVVQ[tiab] OR “Villalta”[tw] OR “Ginsberg”[tw] OR “Brandjes”[tw] OR “Widmer”[tw] OR “Marder” OR “VEINES-QOL”[tw] OR VAS[tw] OR VCSS[tw] OR “Chronic Venous Insufficiency Questionnaire”[tw] OR CIVIQ[tw] OR ”Aberdeen Varicose Vein Questionnaire”[tw] OR AVVQ[tw]) AND (“Meaningful Use”[Mesh] OR improve[tiab] OR enhancement[tiab] OR better[tiab] OR update[tiab] OR “Quality Improvement”[Mesh])) AND ((“vein insufficiency”[tiab] OR “Venous Insufficiency”[Mesh] OR “venous insufficiency”[tiab] OR “vein incompetence”[tiab] OR “venous incompetence”[tiab] OR “vein reflux”[tiab] OR “venous reflux”[tiab]) OR (“post-thrombotic syndrome”[tiab] OR “postthrombotic syndrome”[tiab] OR thrombophlebitis[tiab] OR “post thrombotic”[tiab] OR postthrombosis[tiab] OR postthrombotic[tiab] OR “post thrombosis”[tiab])) |
| OVID MEDLINE= 109 | 12/09/22 | ((Villalta.tw. OR Ginsberg.tw. OR Brandjes.tw. OR Widmer.tw. OR Marder OR VEINES-QOL.tw. OR VAS.tw. OR VCSS.tw. OR "Chronic Venous Insufficiency Questionnaire".tw. OR CIVIQ.tw. OR "Aberdeen Varicose Vein Questionnaire".tw. OR AVVQ.tw. OR Villalta.mp. OR Ginsberg.mp. OR Brandjes.mp. OR Widmer.mp. OR Marder OR VEINES-QOL.mp. OR VAS.mp. OR VCSS.mp. OR "Chronic Venous Insufficiency Questionnaire".mp. OR CIVIQ.mp. OR "Aberdeen Varicose Vein Questionnaire".mp. OR AVVQ.mp.) AND (exp "Meaningful Use"/ OR improve.tw. OR enhancement.tw. OR better.tw. OR update.tw. OR exp "Quality Improvement"/)) AND (("vein insufficiency".tw. OR exp "Venous Insufficiency"/ OR "venous insufficiency".tw. OR "vein incompetence".tw. OR "venous incompetence".tw. OR "vein reflux".tw. OR "venous reflux".tw.) OR ("post-thrombotic syndrome".tw. OR "postthrombotic syndrome".tw. OR thrombophlebitis.tw. OR "post thrombotic".tw. OR postthrombosis.tw. OR postthrombotic.tw. OR "post thrombosis".tw.)) |
| COCHRANE LIBRARY= 92 | 02/09/22 | ((Villalta:ti,ab OR Ginsberg:ti,ab OR Brandjes:ti,ab OR Widmer:ti,ab OR Marder OR VEINES-QOL:ti,ab OR VAS:ti,ab OR VCSS:ti,ab OR "Chronic Venous Insufficiency Questionnaire":ti,ab OR CIVIQ:ti,ab OR "Aberdeen Varicose Vein Questionnaire":ti,ab OR AVVQ:ti,ab OR Villalta:ti,ab,kw OR Ginsberg:ti,ab,kw OR Brandjes:ti,ab,kw OR Widmer:ti,ab,kw OR Marder OR VEINES-QOL:ti,ab,kw OR VAS:ti,ab,kw OR VCSS:ti,ab,kw OR "Chronic Venous Insufficiency Questionnaire":ti,ab,kw OR CIVIQ:ti,ab,kw OR "Aberdeen Varicose Vein Questionnaire":ti,ab,kw OR AVVQ:ti,ab,kw) AND ([mh "Meaningful Use"] OR improve:ti,ab OR enhancement:ti,ab OR better:ti,ab OR update:ti,ab OR [mh "Quality Improvement"])) AND (("vein insufficiency":ti,ab OR [mh "Venous Insufficiency"] OR "venous insufficiency":ti,ab OR "vein incompetence":ti,ab OR "venous incompetence":ti,ab OR "vein reflux":ti,ab OR "venous reflux":ti,ab) OR ("post-thrombotic syndrome":ti,ab OR "postthrombotic syndrome":ti,ab OR thrombophlebitis:ti,ab OR "post thrombotic":ti,ab OR postthrombosis:ti,ab OR postthrombotic:ti,ab OR "post thrombosis":ti,ab)) |
| EMBASE (ELSEVIER)= 237 | 02/09/22 | ((Villalta:ti,ab OR Ginsberg:ti,ab OR Brandjes:ti,ab OR Widmer:ti,ab OR Marder OR VEINES-QOL:ti,ab OR VAS:ti,ab OR VCSS:ti,ab OR 'Chronic Venous Insufficiency Questionnaire':ti,ab OR CIVIQ:ti,ab OR 'Aberdeen Varicose Vein Questionnaire':ti,ab OR AVVQ:ti,ab OR Villalta OR Ginsberg OR Brandjes OR Widmer OR Marder OR VEINES-QOL OR VAS OR VCSS OR 'Chronic Venous Insufficiency Questionnaire' OR CIVIQ OR 'Aberdeen Varicose Vein Questionnaire' OR AVVQ) AND ('Meaningful Use'/exp OR improve:ti,ab OR enhancement:ti,ab OR better:ti,ab OR update:ti,ab OR 'Quality Improvement'/exp)) AND (('vein insufficiency':ti,ab OR 'Venous Insufficiency'/exp OR 'venous insufficiency':ti,ab OR 'vein incompetence':ti,ab OR 'venous incompetence':ti,ab OR 'vein reflux':ti,ab OR 'venous reflux':ti,ab) OR ('post-thrombotic syndrome':ti,ab OR 'postthrombotic syndrome':ti,ab OR thrombophlebitis:ti,ab OR 'post thrombotic':ti,ab OR postthrombosis:ti,ab OR postthrombotic:ti,ab OR 'post thrombosis':ti,ab)) |
| CINAHL (EBSCO)= 20 | 02/09/22 | (((TI Villalta OR AB Villalta) OR (TI Ginsberg OR AB Ginsberg) OR (TI Brandjes OR AB Brandjes) OR (TI Widmer OR AB Widmer) OR Marder OR (TI VEINES-QOL OR AB VEINES-QOL) OR (TI VAS OR AB VAS) OR (TI VCSS OR AB VCSS) OR (TI "Chronic Venous Insufficiency Questionnaire" OR AB "Chronic Venous Insufficiency Questionnaire") OR (TI CIVIQ OR AB CIVIQ) OR (TI "Aberdeen Varicose Vein Questionnaire" OR AB "Aberdeen Varicose Vein Questionnaire") OR (TI AVVQ OR AB AVVQ) OR Villalta OR Ginsberg OR Brandjes OR Widmer OR Marder OR VEINES-QOL OR VAS OR VCSS OR "Chronic Venous Insufficiency Questionnaire" OR CIVIQ OR "Aberdeen Varicose Vein Questionnaire" OR AVVQ) AND ((MH "Meaningful Use+") OR (TI improve OR AB improve) OR (TI enhancement OR AB enhancement) OR (TI better OR AB better) OR (TI update OR AB update) OR (MH "Quality Improvement+"))) AND (((TI "vein insufficiency" OR AB "vein insufficiency") OR (MH "Venous Insufficiency+") OR (TI "venous insufficiency" OR AB "venous insufficiency") OR (TI "vein incompetence" OR AB "vein incompetence") OR (TI "venous incompetence" OR AB "venous incompetence") OR (TI "vein reflux" OR AB "vein reflux") OR (TI "venous reflux" OR AB "venous reflux")) OR ((TI "post-thrombotic syndrome" OR AB "post-thrombotic syndrome") OR (TI "postthrombotic syndrome" OR AB "postthrombotic syndrome") OR (TI thrombophlebitis OR AB thrombophlebitis) OR (TI "post thrombotic" OR AB "post thrombotic") OR (TI postthrombosis OR AB postthrombosis) OR (TI postthrombotic OR AB postthrombotic) OR (TI "post thrombosis" OR AB "post thrombosis"))) |
| SCOPUS (Basic search)= 257 | 02/09/22 | ((Villalta OR Ginsberg OR Brandjes OR Widmer OR Marder OR VEINES-QOL OR VAS OR VCSS OR "Chronic Venous Insufficiency Questionnaire" OR CIVIQ OR "Aberdeen Varicose Vein Questionnaire" OR AVVQ OR Villalta OR Ginsberg OR Brandjes OR Widmer OR Marder OR VEINES-QOL OR VAS OR VCSS OR "Chronic Venous Insufficiency Questionnaire" OR CIVIQ OR "Aberdeen Varicose Vein Questionnaire" OR AVVQ) AND ("Meaningful Use" OR improve OR enhancement OR better OR update OR "Quality Improvement")) AND (("vein insufficiency" OR "Venous Insufficiency" OR "venous insufficiency" OR "vein incompetence" OR "venous incompetence" OR "vein reflux" OR "venous reflux") OR ("post-thrombotic syndrome" OR "postthrombotic syndrome" OR thrombophlebitis OR "post thrombotic" OR postthrombosis OR postthrombotic OR "post thrombosis")) |

1. **Prevention of the post-thrombotic syndrome:** *Need to truly determine if PMT is useful for both iliofemoral DVT and femoropopliteal DVT.

| **Database** | **Date** | **Search Algorithm** |
| --- | --- | --- |
| PUBMED= 264  2000-2022 | 02/09/22 | ((“Venous Thromboembolism”[Mesh] OR “Venous Thrombosis”[Mesh] OR “deep vein thrombosis”[tiab] OR “venous thrombosis”[tiab] OR dvt[tiab] OR chronic[tiab] OR “chronic venous thrombosis”[tiab] OR “chronic vein thrombosis”[tiab]) AND (Thromboembolectomy[tiab] OR “Thrombectomy”[Mesh] OR “mechanical thrombectomy”[tiab] OR “Mechanical Thrombolysis”[Mesh] OR clottriever[tw] OR indigo[tw] OR dovi[tw] OR aspirex[tw] OR thrombolex[tw] OR bashir[tw] OR ekosonic[tw] OR trerotola[tw] OR angiojet[tw] OR trellis[tw] OR Zelante[tw] OR penumbra[tw])) AND (Iliofemoral[tiab] OR femoropopliteal[tiab] OR “Ilio femoral”[tiab] OR “femoro popliteal”[tiab] OR Ilio-femoral[tiab] OR femoro-popliteal[tiab] OR iliocaval[tiab] or iliocaval[tw] OR Iliofemoral[tw] OR femoropopliteal[tw] OR “Ilio femoral”[tw] OR “femoro popliteal”[tw] OR Ilio-femoral[tw] OR femoro-popliteal[tw]) |
| OVID MEDLINE= 267 | 12/09/22 | ((exp "Venous Thromboembolism"/ OR exp "Venous Thrombosis"/ OR "deep vein thrombosis".tw. OR "venous thrombosis".tw. OR dvt.tw. OR chronic.tw. OR "chronic venous thrombosis".tw. OR "chronic vein thrombosis".tw.) AND (Thromboembolectomy.tw. OR exp Thrombectomy/ OR "mechanical thrombectomy".tw. OR exp "Mechanical Thrombolysis"/ OR clottriever.mp. OR indigo.mp. OR dovi.mp. OR aspirex.mp. OR thrombolex.mp. OR bashir.mp. OR ekosonic.mp. OR trerotola.mp. OR angiojet.mp. OR trellis.mp. OR Zelante.mp. OR penumbra.mp.)) AND (Iliofemoral.tw. OR femoropopliteal.tw. OR "Ilio femoral".tw. OR "femoro popliteal".tw. OR Ilio-femoral.tw. OR femoro-popliteal.tw. OR iliocaval.tw. OR iliocaval.mp. OR Iliofemoral.mp. OR femoropopliteal.mp. OR "Ilio femoral".mp. OR "femoro popliteal".mp. OR Ilio-femoral.mp. OR femoro-popliteal.mp.) |
| COCHRANE LIBRARY= 18 | 02/09/22 | (([mh "Venous Thromboembolism"] OR [mh "Venous Thrombosis"] OR "deep vein thrombosis":ti,ab OR "venous thrombosis":ti,ab OR dvt:ti,ab OR chronic:ti,ab OR "chronic venous thrombosis":ti,ab OR "chronic vein thrombosis":ti,ab) AND (Thromboembolectomy:ti,ab OR [mh Thrombectomy] OR "mechanical thrombectomy":ti,ab OR [mh "Mechanical Thrombolysis"] OR clottriever:ti,ab,kw OR indigo:ti,ab,kw OR dovi:ti,ab,kw OR aspirex:ti,ab,kw OR thrombolex:ti,ab,kw OR bashir:ti,ab,kw OR ekosonic:ti,ab,kw OR trerotola:ti,ab,kw OR angiojet:ti,ab,kw OR trellis:ti,ab,kw OR Zelante:ti,ab,kw OR penumbra:ti,ab,kw)) AND (Iliofemoral:ti,ab OR femoropopliteal:ti,ab OR "Ilio femoral":ti,ab OR "femoro popliteal":ti,ab OR Ilio-femoral:ti,ab OR femoro-popliteal:ti,ab OR iliocaval:ti,ab OR iliocaval:ti,ab,kw OR Iliofemoral:ti,ab,kw OR femoropopliteal:ti,ab,kw OR "Ilio femoral":ti,ab,kw OR "femoro popliteal":ti,ab,kw OR Ilio-femoral:ti,ab,kw OR femoro-popliteal:ti,ab,kw) |
| EMBASE (ELSEVIER)= 780 | 02/09/22 | (('Venous Thromboembolism'/exp OR 'Venous Thrombosis'/exp OR 'deep vein thrombosis':ti,ab OR 'venous thrombosis':ti,ab OR dvt:ti,ab OR chronic:ti,ab OR 'chronic venous thrombosis':ti,ab OR 'chronic vein thrombosis':ti,ab) AND (Thromboembolectomy:ti,ab OR Thrombectomy/exp OR 'mechanical thrombectomy':ti,ab OR 'Mechanical Thrombolysis'/exp OR clottriever OR indigo OR dovi OR aspirex OR thrombolex OR bashir OR ekosonic OR trerotola OR angiojet OR trellis OR Zelante OR penumbra)) AND (Iliofemoral:ti,ab OR femoropopliteal:ti,ab OR 'Ilio femoral':ti,ab OR 'femoro popliteal':ti,ab OR Ilio-femoral:ti,ab OR femoro-popliteal:ti,ab OR iliocaval:ti,ab OR iliocaval OR Iliofemoral OR femoropopliteal OR 'Ilio femoral' OR 'femoro popliteal' OR Ilio-femoral OR femoro-popliteal) |
| CINAHL (EBSCO)= 60 | 02/09/22 | (((MH "Venous Thromboembolism+") OR (MH "Venous Thrombosis+") OR (TI "deep vein thrombosis" OR AB "deep vein thrombosis") OR (TI "venous thrombosis" OR AB "venous thrombosis") OR (TI dvt OR AB dvt) OR (TI chronic OR AB chronic) OR (TI "chronic venous thrombosis" OR AB "chronic venous thrombosis") OR (TI "chronic vein thrombosis" OR AB "chronic vein thrombosis")) AND ((TI Thromboembolectomy OR AB Thromboembolectomy) OR (MH Thrombectomy+) OR (TI "mechanical thrombectomy" OR AB "mechanical thrombectomy") OR (MH "Mechanical Thrombolysis+") OR clottriever OR indigo OR dovi OR aspirex OR thrombolex OR bashir OR ekosonic OR trerotola OR angiojet OR trellis OR Zelante OR penumbra)) AND ((TI Iliofemoral OR AB Iliofemoral) OR (TI femoropopliteal OR AB femoropopliteal) OR (TI "Ilio femoral" OR AB "Ilio femoral") OR (TI "femoro popliteal" OR AB "femoro popliteal") OR (TI Ilio-femoral OR AB Ilio-femoral) OR (TI femoro-popliteal OR AB femoro-popliteal) OR (TI iliocaval OR AB iliocaval) OR iliocaval OR Iliofemoral OR femoropopliteal OR "Ilio femoral" OR "femoro popliteal" OR Ilio-femoral OR femoro-popliteal) |
| SCOPUS= 578 | 02/09/22 | (("Venous Thromboembolism" OR "Venous Thrombosis" OR "deep vein thrombosis" OR "venous thrombosis" OR dvt OR chronic OR "chronic venous thrombosis" OR "chronic vein thrombosis") AND (Thromboembolectomy OR Thrombectomy OR "mechanical thrombectomy" OR "Mechanical Thrombolysis" OR clottriever OR indigo OR dovi OR aspirex OR thrombolex OR bashir OR ekosonic OR trerotola OR angiojet OR trellis OR Zelante OR penumbra)) AND (Iliofemoral OR femoropopliteal OR "Ilio femoral" OR "femoro popliteal" OR Ilio-femoral OR femoro-popliteal OR iliocaval OR iliocaval OR Iliofemoral OR femoropopliteal OR "Ilio femoral" OR "femoro popliteal" OR Ilio-femoral OR femoro-popliteal) |

1. **Treatment of C4 to 6, including VU: compression, correction, and surveillance guidelines:** *Come up with a functional deep venous valve and determine in which setting(s) such a valve would be useful.

| **Database** | **Date** | **Search Algorithm** |
| --- | --- | --- |
| PUBMED= 35 | 02/09/22 | ((("Venous Valves"[Mesh] OR valve[tiab] OR "Prostheses and Implants"[Mesh] OR "Venous Valves"[tw] OR "Venous Valve"[tiab] OR "Venous Valve"[tw] NOT aortic[tiab] NOT mitral[tiab] NOT pulmonary[tiab] NOT tricuspid[tiab] NOT heart[tiab] NOT urinary[tiab]) AND (artificial[tiab] OR venovalve[tiab] OR bioprosthetic[tiab] OR artificial[tw] OR venovalve[tw] OR bioprosthetic[tw] OR novel[tiab])) AND (Implementation[tiab] OR use[tiab] OR recommendation[tiab] OR useful[tiab] OR surveillance[tiab] OR implantation[tiab] OR indication[tiab] OR impact[tiab])) AND ("post-thrombotic syndrome"[tiab] OR "postthrombotic syndrome"[tiab] OR thrombophlebitis[tiab] OR "post thrombotic"[tiab] OR postthrombosis[tiab] OR postthrombotic[tiab] OR "post thrombosis"[tiab] OR "Venous Insufficiency"[Mesh] OR "venous insufficiency"[tiab] OR "vein incompetence"[tiab] OR "venous incompetence"[tiab] OR "vein reflux"[tiab] OR "venous reflux"[tiab] OR "venous outflow disease"[tiab] OR "venous outflow obstruction"[tiab] OR "impaired venous outflow"[tiab] OR "venous obstruction"[tiab] OR "vein obstruction"[tiab] OR "venous ulcer"[tiab] OR "vein ulcer"[tiab] OR "varicose ulcer"[tiab] OR "venous ulcers"[tiab] OR "vein ulcers"[tiab] OR "varicose ulcers"[tiab] OR "Venous Thromboembolism"[Mesh] OR "Venous Thrombosis"[Mesh] OR "deep vein thrombosis"[tiab] OR "venous thrombosis"[tiab] OR dvt[tiab] OR "chronic venous thrombosis"[tiab] OR "chronic vein thrombosis"[tiab]) |
| OVID MEDLINE= 63 | 12/09/22 | (((exp "Venous Valves"/ OR valve.tw. OR exp "Prostheses and Implants"/ OR "Venous Valves".mp. OR "Venous Valve".tw. OR "Venous Valve".mp. NOT aortic.tw. NOT mitral.tw. NOT pulmonary.tw. NOT tricuspid.tw. NOT heart.tw. NOT urinary.tw.) AND (artificial.tw. OR venovalve.tw. OR bioprosthetic.tw. OR artificial.mp. OR venovalve.mp. OR bioprosthetic.mp. OR novel.tw.)) AND (Implementation.tw. OR recommendation.tw. OR useful.tw. OR surveillance.tw. OR implantation.tw. OR indication.tw. OR impact.tw.)) AND ("post-thrombotic syndrome".tw. OR "postthrombotic syndrome".tw. OR thrombophlebitis.tw. OR "post thrombotic".tw. OR postthrombosis.tw. OR postthrombotic.tw. OR "post thrombosis".tw. OR exp "Venous Insufficiency"/ OR "venous insufficiency".tw. OR "vein incompetence".tw. OR "venous incompetence".tw. OR "vein reflux".tw. OR "venous reflux".tw. OR "venous outflow disease".tw. OR "venous outflow obstruction".tw. OR "impaired venous outflow".tw. OR "venous obstruction".tw. OR "vein obstruction".tw. OR "venous ulcer".tw. OR "vein ulcer".tw. OR "varicose ulcer".tw. OR "venous ulcers".tw. OR "vein ulcers".tw. OR "varicose ulcers".tw. OR exp "Venous Thromboembolism"/ OR exp "Venous Thrombosis"/ OR "deep vein thrombosis".tw. OR "venous thrombosis".tw. OR dvt.tw. OR "chronic venous thrombosis".tw. OR "chronic vein thrombosis".tw.) |
| COCHRANE LIBRARY= 3866 | 02/09/22 | (((([mh "Venous Valves"] OR valve:ti,ab OR [mh "Prostheses and Implants"] OR "Venous Valves":ti,ab,kw OR "Venous Valve":ti,ab OR "Venous Valve":ti,ab,kw) AND (artificial:ti,ab OR venovalve:ti,ab OR bioprosthetic:ti,ab OR artificial:ti,ab,kw OR venovalve:ti,ab,kw OR bioprosthetic:ti,ab,kw OR novel:ti,ab)) AND (Implementation:ti,ab OR use:ti,ab OR recommendation:ti,ab OR useful:ti,ab OR surveillance:ti,ab OR replacement:ti,ab OR implantation:ti,ab OR indication:ti,ab OR impact:ti,ab OR repair:ti,ab)) AND ((((("post-thrombotic syndrome":ti,ab OR "postthrombotic syndrome":ti,ab OR thrombophlebitis:ti,ab OR "post thrombotic":ti,ab OR postthrombosis:ti,ab OR postthrombotic:ti,ab OR "post thrombosis":ti,ab) OR ([mh "Venous Insufficiency"] OR "venous insufficiency":ti,ab OR "vein incompetence":ti,ab OR "venous incompetence":ti,ab OR "vein reflux":ti,ab OR "venous reflux":ti,ab)) OR ("venous outflow disease":ti,ab OR "venous outflow obstruction":ti,ab OR "impaired venous outflow":ti,ab OR "venous obstruction":ti,ab OR "vein obstruction":ti,ab)) OR ("venous ulcer":ti,ab OR "vein ulcer":ti,ab OR "varicose ulcer":ti,ab OR "venous ulcers":ti,ab OR "vein ulcers":ti,ab OR "varicose ulcers":ti,ab)) OR ([mh "Venous Thromboembolism"] OR [mh "Venous Thrombosis"] OR "deep vein thrombosis":ti,ab OR "venous thrombosis":ti,ab OR dvt:ti,ab OR "chronic venous thrombosis":ti,ab OR "chronic vein thrombosis":ti,ab))) NOT ((aortic:ti,ab OR mitral:ti,ab OR pulmonary:ti,ab OR tricuspid:ti,ab OR heart:ti,ab OR urinary:ti,ab OR orthopedic:ti,ab OR hip:ti,ab OR anesthetic:ti,ab OR knee:ti,ab OR anticoagulant:ti,ab OR antiaggregant:ti,ab OR ventricular:ti,ab OR arterial:ti,ab OR artery:ti,ab OR joint:ti,ab OR cardiac:ti,ab OR pacemaker:ti,ab)) |
| EMBASE (ELSEVIER)= 91 | 02/09/22 | (((('Venous Valves'/exp OR valve:ti,ab OR 'Prostheses and Implants'/exp OR 'Venous Valves' OR 'Venous Valve':ti,ab OR 'Venous Valve') AND (artificial:ti,ab OR venovalve:ti,ab OR bioprosthetic:ti,ab OR artificial OR venovalve OR bioprosthetic OR novel:ti,ab)) AND (Implementation:ti,ab OR use:ti,ab OR recommendation:ti,ab OR useful:ti,ab OR surveillance:ti,ab OR replacement:ti,ab OR implantation:ti,ab OR indication:ti,ab OR impact:ti,ab OR repair:ti,ab)) AND ((((('post-thrombotic syndrome':ti,ab OR 'postthrombotic syndrome':ti,ab OR thrombophlebitis:ti,ab OR 'post thrombotic':ti,ab OR postthrombosis:ti,ab OR postthrombotic:ti,ab OR 'post thrombosis':ti,ab) OR ('Venous Insufficiency'/exp OR 'venous insufficiency':ti,ab OR 'vein incompetence':ti,ab OR 'venous incompetence':ti,ab OR 'vein reflux':ti,ab OR 'venous reflux':ti,ab)) OR ('venous outflow disease':ti,ab OR 'venous outflow obstruction':ti,ab OR 'impaired venous outflow':ti,ab OR 'venous obstruction':ti,ab OR 'vein obstruction':ti,ab)) OR ('venous ulcer':ti,ab OR 'vein ulcer':ti,ab OR 'varicose ulcer':ti,ab OR 'venous ulcers':ti,ab OR 'vein ulcers':ti,ab OR 'varicose ulcers':ti,ab)) OR ('Venous Thromboembolism'/exp OR 'Venous Thrombosis'/exp OR 'deep vein thrombosis':ti,ab OR 'venous thrombosis':ti,ab OR dvt:ti,ab OR 'chronic venous thrombosis':ti,ab OR 'chronic vein thrombosis':ti,ab))) NOT ((aortic:ti,ab OR mitral:ti,ab OR pulmonary:ti,ab OR tricuspid:ti,ab OR heart:ti,ab OR urinary:ti,ab OR orthopedic:ti,ab OR hip:ti,ab OR anesthetic:ti,ab OR knee:ti,ab OR anticoagulant:ti,ab OR antiaggregant:ti,ab OR ventricular:ti,ab OR arterial:ti,ab OR artery:ti,ab OR joint:ti,ab OR cardiac:ti,ab OR pacemaker:ti,ab)) |
| CINAHL (EBSCO)= 17 | 02/09/22 | (((((MH "Venous Valves+") OR (TI valve OR AB valve) OR (MH "Prostheses and Implants+") OR "Venous Valves" OR (TI "Venous Valve" OR AB "Venous Valve") OR "Venous Valve") AND ((TI artificial OR AB artificial) OR (TI venovalve OR AB venovalve) OR (TI bioprosthetic OR AB bioprosthetic) OR artificial OR venovalve OR bioprosthetic OR (TI novel OR AB novel))) AND ((TI Implementation OR AB Implementation) OR (TI use OR AB use) OR (TI recommendation OR AB recommendation) OR (TI useful OR AB useful) OR (TI surveillance OR AB surveillance) OR (TI replacement OR AB replacement) OR (TI implantation OR AB implantation) OR (TI indication OR AB indication) OR (TI impact OR AB impact) OR (TI repair OR AB repair))) AND ((((((TI "post-thrombotic syndrome" OR AB "post-thrombotic syndrome") OR (TI "postthrombotic syndrome" OR AB "postthrombotic syndrome") OR (TI thrombophlebitis OR AB thrombophlebitis) OR (TI "post thrombotic" OR AB "post thrombotic") OR (TI postthrombosis OR AB postthrombosis) OR (TI postthrombotic OR AB postthrombotic) OR (TI "post thrombosis" OR AB "post thrombosis")) OR ((MH "Venous Insufficiency+") OR (TI "venous insufficiency" OR AB "venous insufficiency") OR (TI "vein incompetence" OR AB "vein incompetence") OR (TI "venous incompetence" OR AB "venous incompetence") OR (TI "vein reflux" OR AB "vein reflux") OR (TI "venous reflux" OR AB "venous reflux"))) OR ((TI "venous outflow disease" OR AB "venous outflow disease") OR (TI "venous outflow obstruction" OR AB "venous outflow obstruction") OR (TI "impaired venous outflow" OR AB "impaired venous outflow") OR (TI "venous obstruction" OR AB "venous obstruction") OR (TI "vein obstruction" OR AB "vein obstruction"))) OR ((TI "venous ulcer" OR AB "venous ulcer") OR (TI "vein ulcer" OR AB "vein ulcer") OR (TI "varicose ulcer" OR AB "varicose ulcer") OR (TI "venous ulcers" OR AB "venous ulcers") OR (TI "vein ulcers" OR AB "vein ulcers") OR (TI "varicose ulcers" OR AB "varicose ulcers"))) OR ((MH "Venous Thromboembolism+") OR (MH "Venous Thrombosis+") OR (TI "deep vein thrombosis" OR AB "deep vein thrombosis") OR (TI "venous thrombosis" OR AB "venous thrombosis") OR (TI dvt OR AB dvt) OR (TI "chronic venous thrombosis" OR AB "chronic venous thrombosis") OR (TI "chronic vein thrombosis" OR AB "chronic vein thrombosis")))) NOT (((TI aortic OR AB aortic) OR (TI mitral OR AB mitral) OR (TI pulmonary OR AB pulmonary) OR (TI tricuspid OR AB tricuspid) OR (TI heart OR AB heart) OR (TI urinary OR AB urinary) OR (TI orthopedic OR AB orthopedic) OR (TI hip OR AB hip) OR (TI anesthetic OR AB anesthetic) OR (TI knee OR AB knee) OR (TI anticoagulant OR AB anticoagulant) OR (TI antiaggregant OR AB antiaggregant) OR (TI ventricular OR AB ventricular) OR (TI arterial OR AB arterial) OR (TI artery OR AB artery) OR (TI joint OR AB joint) OR (TI cardiac OR AB cardiac) OR (TI pacemaker OR AB pacemaker))) |
| SCOPUS= 0 | 02/09/22 | (((("Venous Valves" OR valve OR "Prostheses and Implants" OR "Venous Valves" OR "Venous Valve" OR "Venous Valve") AND (artificial OR venovalve OR bioprosthetic OR artificial OR venovalve OR bioprosthetic OR novel)) AND (Implementation OR use OR recommendation OR useful OR surveillance OR replacement OR implantation OR indication OR impact OR repair)) AND ((((("post-thrombotic syndrome" OR "postthrombotic syndrome" OR thrombophlebitis OR "post thrombotic" OR postthrombosis OR postthrombotic OR "post thrombosis") OR ("Venous Insufficiency" OR "venous insufficiency" OR "vein incompetence" OR "venous incompetence" OR "vein reflux" OR "venous reflux")) OR ("venous outflow disease" OR "venous outflow obstruction" OR "impaired venous outflow" OR "venous obstruction" OR "vein obstruction")) OR ("venous ulcer" OR "vein ulcer" OR "varicose ulcer" OR "venous ulcers" OR "vein ulcers" OR "varicose ulcers")) OR ("Venous Thromboembolism" OR "Venous Thrombosis" OR "deep vein thrombosis" OR "venous thrombosis" OR dvt OR "chronic venous thrombosis" OR "chronic vein thrombosis"))) NOT ((aortic OR mitral OR pulmonary OR tricuspid OR heart OR urinary OR orthopedic OR hip OR anesthetic OR knee OR anticoagulant OR antiaggregant OR ventricular OR arterial OR artery OR joint OR cardiac OR pacemaker)) |

1. **Research:** *Biomarkers to help determine which patients with C4 disease will progress to C5/C6 disease and which will not progress.

| **Database** | **Date** | **Search Algorithm** |
| --- | --- | --- |
| PUBMED= 263 | 02/09/22 | ((("Prevention and control"[sh] OR "risk assessment"[Mesh] OR risk*[ti] OR (risk[tiab] AND (assess*[tiab] OR evaluat*[tiab]) OR Stratifying[tiab] OR stratification*[tiab] OR predict[tiab] OR "Disease Progression"[Mesh]) AND (Implementation[tiab] OR use[tiab] OR recommendation[tiab] OR useful[tiab] OR surveillance[tiab] OR indication[tiab] OR impact[tiab] OR assessment[tiab] OR evaluation[tiab])) AND ("Biomarkers"[tiab] OR "Biological Factors"[Mesh] OR "Phenotype"[Mesh] OR "Genetic Markers"[Mesh])) AND (((("Varicose Veins"[Mesh] OR "May-Thurner Syndrome"[Mesh] OR "May-Thurner Syndrome"[tiab] OR "Thrombophlebitis"[Mesh] OR "varicose vein"[tiab] OR "varicose veins"[tiab] OR varicosity[tiab] OR "venous stasis"[tiab]) OR ("venous ulcer"[tiab] OR "vein ulcer"[tiab] OR "varicose ulcer"[tiab] OR "venous ulcers"[tiab] OR "vein ulcers"[tiab] OR "varicose ulcers"[tiab])) OR ("Venous Thromboembolism"[Mesh] OR "Venous Thrombosis"[Mesh] OR "deep vein thrombosis"[tiab] OR "venous thrombosis"[tiab] OR dvt[tiab] OR "chronic venous thrombosis"[tiab] OR "chronic vein thrombosis"[tiab])) OR ("post-thrombotic syndrome"[tiab] OR "postthrombotic syndrome"[tiab] OR thrombophlebitis[tiab] OR "post thrombotic"[tiab] OR postthrombosis[tiab] OR postthrombotic[tiab] OR "post thrombosis"[tiab]))) NOT ((aortic[tiab] OR mitral[tiab] OR pulmonary[tiab] OR tricuspid[tiab] OR heart[tiab] OR urinary[tiab] OR orthopedic[tiab] OR hip[tiab] OR anesthetic[tiab] OR knee[tiab] OR anticoagulant[tiab] OR antiaggregant[tiab] OR ventricular[tiab] OR arterial[tiab] OR artery[tiab] OR joint[tiab] OR cardiac[tiab] OR pacemaker[tiab] OR cancer[tiab] OR infections[Mesh] OR sepsis[tiab] OR septic[tiab] OR tumor[tiab] OR brain[tiab] cerebral[tiab] OR fracture[tiab] OR "Peripheral Vascular Diseases"[Mesh] OR "acute coronary syndrome"[Mesh] OR Neoplasms[Mesh])) |
| OVID MEDLINE= 297 | 12/09/22 | ((("Prevention & Control".fs. OR exp "risk assessment"/ OR risk*.ti. OR (risk.tw. AND (assess*.tw. OR evaluat*.tw.) OR Stratifying.tw. OR stratification*.tw. OR predict.tw. OR exp "Disease Progression"/) AND (Implementation.tw. OR recommendation.tw. OR useful.tw. OR surveillance.tw. OR indication.tw. OR impact.tw. OR assessment.tw. OR evaluation.tw.)) AND (Biomarkers.tw. OR exp "Biological Factors"/ OR exp Phenotype/ OR exp "Genetic Markers"/)) AND ((((exp "Varicose Veins"/ OR exp "May-Thurner Syndrome"/ OR "May-Thurner Syndrome".tw. OR exp Thrombophlebitis/ OR "varicose vein".tw. OR "varicose veins".tw. OR varicosity.tw. OR "venous stasis".tw.) OR ("venous ulcer".tw. OR "vein ulcer".tw. OR "varicose ulcer".tw. OR "venous ulcers".tw. OR "vein ulcers".tw. OR "varicose ulcers".tw.)) OR (exp "Venous Thromboembolism"/ OR exp "Venous Thrombosis"/ OR "deep vein thrombosis".tw. OR "venous thrombosis".tw. OR dvt.tw. OR "chronic venous thrombosis".tw. OR "chronic vein thrombosis".tw.)) OR ("post-thrombotic syndrome".tw. OR "postthrombotic syndrome".tw. OR thrombophlebitis.tw. OR "post thrombotic".tw. OR postthrombosis.tw. OR postthrombotic.tw. OR "post thrombosis".tw.))) NOT ((aortic.tw. OR mitral.tw. OR pulmonary.tw. OR tricuspid.tw. OR heart.tw. OR urinary.tw. OR orthopedic.tw. OR hip.tw. OR anesthetic.tw. OR knee.tw. OR anticoagulant.tw. OR antiaggregant.tw. OR ventricular.tw. OR arterial.tw. OR artery.tw. OR joint.tw. OR cardiac.tw. OR pacemaker.tw. OR cancer.tw. OR exp infections/ OR sepsis.tw. OR septic.tw. OR tumor.tw. OR "brain cerebral".tw. OR fracture.tw. OR exp "Peripheral Vascular Diseases"/ OR exp "acute coronary syndrome"/ OR exp Neoplasms/)) |
| COCHRANE LIBRARY= 95 | 02/09/22 | ((([mh /PC] OR [mh "risk assessment"] OR risk*:ti OR (risk:ti,ab AND (assess*:ti,ab OR evaluat*:ti,ab) OR Stratifying:ti,ab OR stratification*:ti,ab OR predict:ti,ab OR [mh "Disease Progression"]) AND (Implementation:ti,ab OR use:ti,ab OR recommendation:ti,ab OR useful:ti,ab OR surveillance:ti,ab OR indication:ti,ab OR impact:ti,ab OR assessment:ti,ab OR evaluation:ti,ab)) AND (Biomarkers:ti,ab OR [mh "Biological Factors"] OR [mh Phenotype] OR [mh "Genetic Markers"])) AND (((([mh "Varicose Veins"] OR [mh "May-Thurner Syndrome"] OR "May-Thurner Syndrome":ti,ab OR [mh Thrombophlebitis] OR "varicose vein":ti,ab OR "varicose veins":ti,ab OR varicosity:ti,ab OR "venous stasis":ti,ab) OR ("venous ulcer":ti,ab OR "vein ulcer":ti,ab OR "varicose ulcer":ti,ab OR "venous ulcers":ti,ab OR "vein ulcers":ti,ab OR "varicose ulcers":ti,ab)) OR ([mh "Venous Thromboembolism"] OR [mh "Venous Thrombosis"] OR "deep vein thrombosis":ti,ab OR "venous thrombosis":ti,ab OR dvt:ti,ab OR "chronic venous thrombosis":ti,ab OR "chronic vein thrombosis":ti,ab)) OR ("post-thrombotic syndrome":ti,ab OR "postthrombotic syndrome":ti,ab OR thrombophlebitis:ti,ab OR "post thrombotic":ti,ab OR postthrombosis:ti,ab OR postthrombotic:ti,ab OR "post thrombosis":ti,ab))) NOT ((aortic:ti,ab OR mitral:ti,ab OR pulmonary:ti,ab OR tricuspid:ti,ab OR heart:ti,ab OR urinary:ti,ab OR orthopedic:ti,ab OR hip:ti,ab OR anesthetic:ti,ab OR knee:ti,ab OR anticoagulant:ti,ab OR antiaggregant:ti,ab OR ventricular:ti,ab OR arterial:ti,ab OR artery:ti,ab OR joint:ti,ab OR cardiac:ti,ab OR pacemaker:ti,ab OR cancer:ti,ab OR [mh infections] OR sepsis:ti,ab OR septic:ti,ab OR tumor:ti,ab OR "brain cerebral":ti,ab OR fracture:ti,ab OR [mh "Peripheral Vascular Diseases"] OR [mh "acute coronary syndrome"] OR [mh Neoplasms])) |
| EMBASE (ELSEVIER)= 92 | 02/09/22 | ((("Prevention & Control" OR 'risk assessment'/exp OR risk*:ti OR (risk:ti,ab AND (assess*:ti,ab OR evaluat*:ti,ab) OR Stratifying:ti,ab OR stratification*:ti,ab OR predict:ti,ab OR 'Disease Progression'/exp) AND (Implementation:ti,ab OR use:ti,ab OR recommendation:ti,ab OR useful:ti,ab OR surveillance:ti,ab OR indication:ti,ab OR impact:ti,ab OR assessment:ti,ab OR evaluation:ti,ab)) AND (Biomarkers:ti,ab OR 'Biological Factors'/exp OR Phenotype/exp OR 'Genetic Markers'/exp)) AND (((('Varicose Veins'/exp OR 'May-Thurner Syndrome'/exp OR 'May-Thurner Syndrome':ti,ab OR Thrombophlebitis/exp OR 'varicose vein':ti,ab OR 'varicose veins':ti,ab OR varicosity:ti,ab OR 'venous stasis':ti,ab) OR ('venous ulcer':ti,ab OR 'vein ulcer':ti,ab OR 'varicose ulcer':ti,ab OR 'venous ulcers':ti,ab OR 'vein ulcers':ti,ab OR 'varicose ulcers':ti,ab)) OR ('Venous Thromboembolism'/exp OR 'Venous Thrombosis'/exp OR 'deep vein thrombosis':ti,ab OR 'venous thrombosis':ti,ab OR dvt:ti,ab OR 'chronic venous thrombosis':ti,ab OR 'chronic vein thrombosis':ti,ab)) OR ('post-thrombotic syndrome':ti,ab OR 'postthrombotic syndrome':ti,ab OR thrombophlebitis:ti,ab OR 'post thrombotic':ti,ab OR postthrombosis:ti,ab OR postthrombotic:ti,ab OR 'post thrombosis':ti,ab))) NOT ((aortic:ti,ab OR mitral:ti,ab OR pulmonary:ti,ab OR tricuspid:ti,ab OR heart:ti,ab OR urinary:ti,ab OR orthopedic:ti,ab OR hip:ti,ab OR anesthetic:ti,ab OR knee:ti,ab OR anticoagulant:ti,ab OR antiaggregant:ti,ab OR ventricular:ti,ab OR arterial:ti,ab OR artery:ti,ab OR joint:ti,ab OR cardiac:ti,ab OR pacemaker:ti,ab OR cancer:ti,ab OR infections/exp OR sepsis:ti,ab OR septic:ti,ab OR tumor:ti,ab OR 'brain cerebral':ti,ab OR fracture:ti,ab OR 'Peripheral Vascular Diseases'/exp OR 'acute coronary syndrome'/exp OR Neoplasms/exp)) |
| CINAHL (EBSCO)= | 02/09/22 | ((("Prevention & Control" OR (MH "risk assessment+") OR TI risk* OR ((TI risk OR AB risk) AND ((TI assess* OR AB assess*) OR (TI evaluat* OR AB evaluat*)) OR (TI Stratifying OR AB Stratifying) OR (TI stratification* OR AB stratification*) OR (TI predict OR AB predict) OR (MH "Disease Progression+")) AND ((TI Implementation OR AB Implementation) OR (TI use OR AB use) OR (TI recommendation OR AB recommendation) OR (TI useful OR AB useful) OR (TI surveillance OR AB surveillance) OR (TI indication OR AB indication) OR (TI impact OR AB impact) OR (TI assessment OR AB assessment) OR (TI evaluation OR AB evaluation))) AND ((TI Biomarkers OR AB Biomarkers) OR (MH "Biological Factors+") OR (MH Phenotype+) OR (MH "Genetic Markers+"))) AND (((((MH "Varicose Veins+") OR (MH "May-Thurner Syndrome+") OR (TI "May-Thurner Syndrome" OR AB "May-Thurner Syndrome") OR (MH Thrombophlebitis+) OR (TI "varicose vein" OR AB "varicose vein") OR (TI "varicose veins" OR AB "varicose veins") OR (TI varicosity OR AB varicosity) OR (TI "venous stasis" OR AB "venous stasis")) OR ((TI "venous ulcer" OR AB "venous ulcer") OR (TI "vein ulcer" OR AB "vein ulcer") OR (TI "varicose ulcer" OR AB "varicose ulcer") OR (TI "venous ulcers" OR AB "venous ulcers") OR (TI "vein ulcers" OR AB "vein ulcers") OR (TI "varicose ulcers" OR AB "varicose ulcers"))) OR ((MH "Venous Thromboembolism+") OR (MH "Venous Thrombosis+") OR (TI "deep vein thrombosis" OR AB "deep vein thrombosis") OR (TI "venous thrombosis" OR AB "venous thrombosis") OR (TI dvt OR AB dvt) OR (TI "chronic venous thrombosis" OR AB "chronic venous thrombosis") OR (TI "chronic vein thrombosis" OR AB "chronic vein thrombosis"))) OR ((TI "post-thrombotic syndrome" OR AB "post-thrombotic syndrome") OR (TI "postthrombotic syndrome" OR AB "postthrombotic syndrome") OR (TI thrombophlebitis OR AB thrombophlebitis) OR (TI "post thrombotic" OR AB "post thrombotic") OR (TI postthrombosis OR AB postthrombosis) OR (TI postthrombotic OR AB postthrombotic) OR (TI "post thrombosis" OR AB "post thrombosis")))) NOT (((TI aortic OR AB aortic) OR (TI mitral OR AB mitral) OR (TI pulmonary OR AB pulmonary) OR (TI tricuspid OR AB tricuspid) OR (TI heart OR AB heart) OR (TI urinary OR AB urinary) OR (TI orthopedic OR AB orthopedic) OR (TI hip OR AB hip) OR (TI anesthetic OR AB anesthetic) OR (TI knee OR AB knee) OR (TI anticoagulant OR AB anticoagulant) OR (TI antiaggregant OR AB antiaggregant) OR (TI ventricular OR AB ventricular) OR (TI arterial OR AB arterial) OR (TI artery OR AB artery) OR (TI joint OR AB joint) OR (TI cardiac OR AB cardiac) OR (TI pacemaker OR AB pacemaker) OR (TI cancer OR AB cancer) OR (MH infections+) OR (TI sepsis OR AB sepsis) OR (TI septic OR AB septic) OR (TI tumor OR AB tumor) OR (TI "brain cerebral" OR AB "brain cerebral") OR (TI fracture OR AB fracture) OR (MH "Peripheral Vascular Diseases+") OR (MH "acute coronary syndrome+") OR (MH Neoplasms+))) |
| SCOPUS=  9010 | 02/09/22 | Neoplasms ( ALL ( ( ( prevention OR control OR "risk assessment" OR risk* OR risk OR assess* OR evaluat* OR stratifying OR stratification* OR predict OR "disease progression" ) AND ( implementation OR recommendation OR useful OR surveillance OR indication OR impact OR assessment OR evaluation ) ) ) AND ALL ( biomarkers OR "biological factors" OR phenotype OR "genetic markers" ) AND ALL ( "varicose veins" OR "may-thurner syndrome" OR "may-thurner syndrome" OR thrombophlebitis OR "varicose vein" OR "varicose veins" OR varicosity OR "venous stasis" OR "venous ulcer" OR "vein ulcer" OR "varicose ulcer" OR "venous ulcers" OR "vein ulcers" OR "varicose ulcers" OR "venous thromboembolism" OR "venous thrombosis" OR "deep vein thrombosis" OR "venous thrombosis" OR dvt OR "chronic venous thrombosis" OR "chronic vein thrombosis" OR "post-thrombotic syndrome" OR "postthrombotic syndrome" OR thrombophlebitis OR "post thrombotic" OR postthrombosis OR postthrombotic OR "post thrombosis" ) AND NOT TITLE-ABS-KEY ( aortic OR mitral OR pulmonary OR tricuspid OR heart OR urinary OR orthopedic OR hip OR anesthetic OR knee OR anticoagulant OR antiaggregant OR ventricular OR arterial OR artery OR joint OR cardiac OR pacemaker OR cancer OR infections OR sepsis OR septic OR tumor OR "brain cerebral" OR fracture OR "peripheral vascular diseases" OR "acute coronary syndrome" OR neoplasms ) ) |

**Supplemental Data**

***Included articles reference list.***

***AIM 1***

1. Lattimer C, Kalodiki E, Azzam M, Geroulakos G. Responsiveness of individual questions from the venous clinical severity score and the Aberdeen varicose vein questionnaire. Phlebology / Venous Forum of the Royal Society of Medicine. 2014;29(1):43‐51.
2. Santiago F, Albernaz D, Zignani F, Silva A, Barroso G, Chi Y. Multi-center evaluation of the aberdeen varicose vein questionnaire APP as a new tool to evaluate quality of life in venous disease. Phlebology / Venous Forum of the Royal Society of Medicine. 2016;31(2):18‐.
3. Engeseth M, Enden T, Sandset P, Wik H. Limitations of the Villalta scale in diagnosing post-thrombotic syndrome. Thrombosis research. 2019;184:62‐66.
4. Kahn S, Gu CS, Galanaud JP, Kearon C, Vedantham S. Exploring alternative approaches of using the villalta scale to capture the post-thrombotic syndrome: a sub-analysis of the attract trial. Research and practice in thrombosis and haemostasis. 2020;4:1097‐1098.
5. Lee A, Gu CS, Vedantham S, Kearon C, Blostein M, Kahn S. Comparison of Two Clinical Scales to Assess the Post-Thrombotic Syndrome: secondary Analysis of a Multicenter Randomized Trial of Pharmacomechanical Catheter-Directed Thrombolysis for Deep Vein Thrombosis. Thrombosis research. 2019;182:2‐3.
6. Schul M, Mallick R, Wright D. Patient reported symptoms (VVSymQ®) predict functional improvement post-endovenous chemical ablation. Phlebology / Venous Forum of the Royal Society of Medicine. 2016;31(2):11‐13.
7. Morris R. Quality of Life Outcomes after Iliac Vein Stenting for Chronic Deep Venous Obstruction: A Tertiary Centre Experience Using The VEINES-QoL/Sym. J Vasc Surg Venous Lymphatic Disord. 2022;10(2):574.
8. Smith J, Thornhill D, Goldenberg NA, Brandao L, Knapp-Clevenger R, Warren BB, et al. Validation of Outcome Instruments for Pediatric Postthrombotic Syndrome: Introducing the Peds-VEINES-QOL, a New Health-Related Quality of Life Instrument. Thromb Haemost. 2021;121(10):1367–75.
9. Lee A, Gu CS, Vedantham S, Kearon C, Blostein M, Kahn SR. Performance of two clinical scales to assess quality of life in patients with post-thrombotic syndrome. J Vasc Surg Venous Lymphat Disord. 2021;9(5):1257-1265.e2.
10. Catarinella FS, Nieman FHM, de Wolf MAF, Toonder IM, de Graaf R, Wittens CHA. Quality-of-life in interventionally treated patients with post-thrombotic syndrome. PHLEBOLOGY. 2015;30(1):89–94.
11. Ortega-Santana F, Liminana JM, Ruano F, Ortega-Centol A, Palomino-Martin A, Jimenez F. The influence of the CIVIQ dimensions on quality of life of patients with primary superficial venous incompetence. Eur J Vasc Endovasc Surg. 2014;48(4):452–8.
12. Allegra C, Antignani PL. Quality of live as measured by the CIVQ 20. Questionnaire following oral mesoglycan treatment of patients with chronic venous disease. Int Angiol. 2014;33(5):407–18.
13. Kaneoka Y, Maeda A, Sugimoto M, Isogai M, Ishibashi H. Quality of life and the venous function of the lower limb after harvest of autologous external iliac vein grafts: a clinical follow-up study. SURG TODAY. 2013;43(11):1254–60.
14. Tan MKH, Sutanto SA, Onida S, Davies AH. The Relationship Between Vein Diameters, Clinical Severity, and Quality of Life: A Systematic Review. Eur J Vasc Endovasc Surg. 2019;57(6):851–7.
15. Kuet ML, Lane TR, Anwar MA, Davies AH. Comparison of disease-specific quality of life tools in patients with chronic venous disease. Phlebology. 2014;29(10):648–53.
16. Andreozzi GM, Cordova RM, Scomparin A, Martini R, D’Eri A, Andreozzi F. Quality of life in chronic venous insufficiency. An Italian pilot study of the Triveneto Region. Int Angiol. 2005;24(3):272–7.
17. Huang Y, Gloviczki P. Relationships between duplex findings and quality of life in long-term follow-up of patients treated for chronic venous disease. Phlebology. 2016;31(1):88–98.
18. Marston WA, Vasquez MA, Lurie F, Wakefield TW, Rabe E, Shortell CK, et al. Multicenter assessment of the repeatability and reproducibility of the revised Venous Clinical Severity Score (rVCSS). J Vasc Surg Venous Lymphat Disord. 2013;1(3):219–24.

***AIM 2***

1. Liu G, Li W, Lu X, Jiang M. Comparison of direct iliofemoral stenting with staged stenting after AngioJet rheolytic thrombectomy in patients with acute deep vein thrombosis. Journal of vascular surgery. 2017;(6):25S.
2. Lichtenberg M. Single session mechanical thrombectomy in iliofemoral dvt patients: interim analysis of vetex study. Vasa - european journal of vascular medicine. 2020;49:23‐.
3. Bozok Ş, Tayfur K. Initial experience with a new pharmacomechanical thrombectomy device for deep venous thrombosis: Report of 91 cases. PHLEBOLOGY. 2019;34(10):707–14.
4. Kim MS, Park HS, Hyun D, Cho SK, Park KB, Do YS. Factors associated with the development of post-thrombotic syndrome in patients with iliofemoral deep venous thrombosis who underwent catheter-directed thrombolysis. PHLEBOLOGY. 2020;35(9):672–8.
5. Comerota AJ, Kearon C, Gu CS, Julian JA, Goldhaber SZ, Kahn SR, et al. Endovascular Thrombus Removal for Acute Iliofemoral Deep Vein Thrombosis: Analysis From a Stratified Multicenter Randomized Trial. CIRCULATION. 2019;139(9):1162–73.
6. Vedantham S, Salter A, Lancia S, Lewis L, Thukral S, Kahn SR. Clinical Outcomes of a Pharmacomechanical Catheter-Directed Venous Thrombolysis Strategy that Included Rheolytic Thrombectomy in a Multicenter Randomized Trial. J VASC INTERVENT RADIOL. 2021;32(9):1296–1296.
7. Argyriou CM, Georgakarakos E, Koutsoumpelis A, Tasopoulou KM, Popidis S, Georgiadis GS. Endovascular Management of Chronic Iliofemoral Venous Thombosis-A Systematic Review and Meta-analysis. Eur J Vasc Endovasc Surg. 2019;58(6):e359–60.
8. Broderick C, Watson L, Armon MP. Thrombolytic strategies versus standard anticoagulation for acute deep vein thrombosis of the lower limb. Cochrane Database Syst Rev [Internet]. 2021;2021(1).
9. Budak AB, Gunaydin S, Gunertem OE, Tumer NB, Ozisik K. Hybridization of pharmacomechanical thrombectomy with catheter-directed thrombolysis: Improving management of acute iliofemoral deep vein thrombosis. Innov Technol Tech Cardiothorac Vasc Surg. 2018;13:S45.
10. Cakir V, Gülcü A, Kucuk B, Capar AE, Gencpinar T, Karabay O, et al. Early results of percutaneous aspiration thrombectomy vs. anticoagulation in acute iliofemoral venous trombosis: A randomised clinical trial. Cardiovasc Intervent Radiol. 2011;34:524.
11. Casey ET, Murad MH, Meissner M, Garcia MZ, Elamin MB, Shi Q, et al. Treatment of acute iliofemoral deep vein thrombosis: A systematic review and meta-analysis. J Vasc Surg. 2009;49(5):40S-41S.
12. Dopheide JF, Sebastian T, Engelberger RP, Haine A, Kucher N. Early clinical outcomes of a novel rheolytic directional thrombectomy technique for patients with iliofemoral deep vein thrombosis. Vasa Euro J Vasc Med. 2018;47(1):56–62.
13. Enden T, Haig Y, Kløw NE, Slagsvold CE, Sandvik L, Ghanima W, et al. Long-term outcome after additional catheter-directed thrombolysis versus standard treatment for acute iliofemoral deep vein thrombosis (the CaVenT study): A randomised controlled trial. Lancet. 2012;379(9810):31–8.
14. Engelberger RP, Fahrni J, Willenberg T, Baumann F, Spirk D, Diehm N, et al. Fixed low-dose ultrasound-assisted catheter-directed thrombolysis followed by routine stenting of residual stenosis for acute ilio-femoral deep-vein thrombosis. Thromb Haemost. 2014;111(6):1153–60.
15. Farsad K, Kapoor BS, Fidelman N, Cain TR, Caplin DM, Eldrup-Jorgensen J, et al. ACR Appropriateness Criteria® Radiologic Management of Iliofemoral Venous Thrombosis. J Am Coll Radiol. 2020;17(5):S255–64.
16. Gaballah M, Shi J, Kukreja K, Obi C, Sola J, Raffini L, et al. Endovascular thrombolysis in management of iliofemoral thrombosis in children: A multiinstitutional experience. J Vasc Intervent Radiol. 2015;26(2):S43–4.
17. Gaballah M, Obi C, Sola J, Raffini L, Keller MS, Krishnamurthy G, et al. Catheter-directed and mechanical thrombolysis for management of lower extremity iliofemoral deep venous thrombosis: A single pediatric institutional experience. J Vasc Intervent Radiol. 2013;24(4):S28–9.
18. Garcia M, Sterling K, Jaff M, Ouriel K, Weinberg I, Kahn S, et al. ACCESS PTS Study: ACCElerated thrombolySiS for post-Thrombotic syndrome using the acoustic pulse thrombolysis EkoSonic® endovascular system: Midterm results of a multicenter study. J Vasc Intervent Radiol. 2018;29(4):S151.
19. Granobles-Molina JD, Tobar JF, Chaves JC, Merchán-Galvis ÁMa. Pharmacomechanical catheter-directed management of deep venous thrombosis: Case series. Tratamiento farmacomecánico dirigido por catéter de la trombosis venosa profunda: Informe de casos. 2020;48(4):107–16.
20. Guo J, Gu Y. Whole progress management of acute iliofemoral venous thrombotic disease treated by AngioJet$sup$®$/sup$ combined with Wallstent$sup$®$/sup$. Int Angiol. 2018;37:25.
21. Hager E, Yuo T, Avgerinos E, Naddaf A, Jeyabalan G, Marone L, et al. Anatomic and functional outcomes of pharmacomechanical and catheter-directed thrombolysis of iliofemoral deep venous thrombosis. J Vasc Surg Venous Lymphatic Disord. 2014;2(3):246–52.
22. Hager ES, Yuo T, Tahara R, Dillavou E, Al-Khoury G, Marone L, et al. Outcomes of endovascular intervention for May-Thurner syndrome. J Vasc Surg Venous Lymphatic Disord. 2013;1(3):270–5.
23. Huang T, Ding W, Chen Z, Yin Y, Yu J, Jin Y, et al. Comparison of Pharmacomechanical Catheter-Directed Thrombolysis versus Catheter-Directed Thrombolysis for the Treatment of Acute Iliofemoral Deep Vein Thrombosis: Measures of Long-Term Clinical Outcome and Quality of Life. Ann Vasc Surg. 2021;76:436–42.
24. Jia Z, Tu J, Zhao J, Ren B, Tian F, Wang K, et al. Aspiration thrombectomy using a large-size catheter for acute lower extremity deep vein thrombosis. J Vasc Surg Venous Lymphatic Disord. 2016;4(2):167–71.
25. Jin W, Yu G, Huang J, Lu K, Huang C. Timing of Endovascular Interventions for Iliac Vein Compression Syndrome With Thrombus. Clin Appl Thromb Hemost [Internet]. 2021;27.
26. Kahn SR, Comerota AJ, Cushman M, Evans NS, Ginsberg JS, Goldenberg NA, et al. The postthrombotic syndrome: Evidence-based prevention, diagnosis, and treatment strategies: A scientific statement from the American heart association. Circulation. 2014;130(18):1636–61.
27. Kim MS, Park HS, Hyun D, Cho SK, Park KB, Shin SW, et al. Predictors of post-thrombotic syndrome in patients with iliofemoral deep venous thrombosis who underwent catheter-directed thrombolysis. Cardiovasc Intervent Radiol. 2019;42(3):S498.
28. Kirkham EN, Hickson G, Ramnarine R, Cooper DG. A ten-year experience of thrombolysis for lower limb ileo-femoral DVTs. Ital J Vasc Endovasc Surg. 2021;28(4):105–12.
29. Lee JJ, Al-Jubouri M, Acino R, Comerota AJ, Lurie F. Role of coexisting contralateral primary venous disease in development of post-thrombotic syndrome following catheter-based treatment of iliofemoral deep venous thrombosis Presented at the Twenty-sixth Annual Meeting of the American Venous Forum, New Orleans, La, February 19-21, 2014. J Vasc Surg Venous Lymphatic Disord. 2015;3(4):354–7.
30. Lee JK, Kim KY, Byun SJ. Safety and efficacy of aspiration thrombectomy or pharmacomechanical thrombectomy after catheter-directed thrombolysis for the treatment of acute iliofemoral deep vein thrombosis. Vasc Spec Internat. 2020;36(3):144–50.
31. Lichtenberg M, Stahlhoff WF, Özkapi A, de Graaf R, Breuckmann F. Safety, procedural success and outcome of the aspirex®s endovascular thrombectomy system in the treatment of iliofemoral deep vein thrombosis – data from the arnsberg aspirex registry. Vasa Euro J Vasc Med. 2019;48(4):341–6.
32. Madsen CP, Gesla J, Vijdea RL, Serifi MA, Christensen JK, Houlind K. Results of catheter-directed thrombolysis for acute ilio-femoral deep venous thrombosis – A retrospective cohort study. JRSM Cardiovascular Dis [Internet]. 2018;7.
33. Mastoris I, Kokkinidis DG, Bikakis I, Archontakis-Barakakis P, Papanastasiou CA, Jonnalagadda AK, et al. Catheter-directed thrombolysis vs. anticoagulation for the prevention and treatment of post-thrombotic syndrome in deep vein thrombosis: An updated systematic review and meta-analysis of randomized trials. Phlebology. 2019;34(10):675–82.
34. Mehdipour M, Sharifi M, Smith G, Starling M, Tarlian H. Reduction of post-thrombotic syndrome in acute deep venous thrombosis by power pulse spray and angiojet thrombectomy. Catheter Cardiovasc Interventions. 2009;73:S43.
35. Ming ZB, Li WD, Yuan RF, Li XQ, Ding WB. Effectiveness of catheter directed thrombolysis and stent implantation on iliofemoral vein thrombosis caused by iliac vein compression. J Thromb Thrombolysis. 2017;44(2):254–60.
36. Notten P, ten Cate-Hoek AJ, Arnoldussen CWKP, Strijkers RHW, de Smet AAEA, Tick LW, et al. Ultrasound-accelerated catheter-directed thrombolysis versus anticoagulation for the prevention of post-thrombotic syndrome (CAVA): a single-blind, multicentre, randomised trial. Lancet Haematol. 2020;7(1):e40–9.
37. Oliveira FAC, Campedelli FL, De Sousa Amorelli CE, Heckmann DD, Figueiredo MBF, De Carvalho GM. Pharmacomechanical catheter-directed therapy in the treatment of deep venous thrombosis: Series of cases. Vasc Endovasc Surg. 2018;52(8):S56.
38. Patra S. Catheter directed thrombolysis along with mechanical thromboaspiration in the management of proximal lower limb deep venous thrombosis-A prospective study. Eur Heart J Acute Cardiovasc Care. 2014;3(2):210.
39. Patra S, Nagesh CM, Reddy B, Srinivas BC, Manjunath CN. Catheter directed thrombolysis in the management of proximal lower limb deep venous thrombosis-a prospective study with 6-month follow-up. Indian Heart J. 2014;66:S93.
40. Patra S, Nagesh CM, Reddy B, Srinivas BC, Manjunath CN. Outcome of venous stenting following catheter directed thrombolysis for acute proximal lower limb venous thrombosis: 1-year follow-up. Indian Heart J. 2014;66:S93.
41. Patra S, Nagesh CM, Reddy B, Srinivas BC, Manjunath CN. Catheter directed thrombolysis along with mechanical thromboaspiration versus anticoagulation alone in the management of lower limb deep venous thrombosis-a comparative study. Indian Heart J. 2014;66:S93–4.
42. Peng Y, Lou W, Gu J, He X, Chen G, Chen L, et al. Modified manual aspiration thrombectomy for the treatment of acute iliofemroal deep vein thrombosis: A comparative study. J Intervent Radiol. 2018;27(6):510–5.
43. Pouncey A, Babigumira J, Johnson O, Black S. Cost Utility Analysis of Treatment Modalities for Iliofemoral Deep Venous Thrombosis: Oral Anticoagulation vs. Catheter Directed Thrombolysis and Pharmacomechanical Thrombectomy. Eur J Vasc Endovasc Surg. 2019;58(6):e700.
44. Raval M, Rajendran S, Stephen E. The Outcome of Catheter-Directed Thrombolysis in COVID-19-Associated Deep Vein Thrombosis. Vasc Endovasc Surg. 2022;56(3):258–62.
45. Roček M. Thrombolysis. Cardiovasc Intervent Radiol. 2016;39(3):S140–1.
46. Rodoplu O, Yildiz CE, Oztas DM, Beyaz MO, Ulukan MO, Unal O, et al. The efficacy of rotational pharmaco-mechanical thrombectomy in patients with acute iliofemoral deep vein thrombosis: Is the standard treatment of deep vein thrombosis changing? Phlebology. 2021;36(2):119–26.
47. Rodriguez LE, Aponte-Rivera F, Figueroa Vicente R, Martinez Trabal JL. Hybrid operative thrombectomy for the treatment of symptomatic iliofemoral deep venous thrombosis: Initial experience and midterm results. J Vasc Surg Venous Lymphatic Disord. 2015;3(1):131.
48. Sharifi M, Freeman W, Bay C, Sharifi M, Schwartz F. Low incidence of post-thrombotic syndrome in patients treated with new oral anticoagulants and percutaneous endovenous intervention for lower extremity deep venous thrombosis. Vasc Med. 2015;20(2):112–6.
49. Sharifi M, Mehdipour M, Berkovits A, Smith G. Percutaneous endovenous intervention reduces post thrombotic syndrome and recurrent venous thromboembolic disease in acute deep venous thrombosis. J Am Coll Cardiol. 2010;55(10):A180.E1686.
50. Tichelaar VYIG, Brodin EE, Vik A, Isaksen T, Skjeldestad FE, Kumar S, et al. A Retrospective Comparison of Ultrasound-Assisted Catheter-Directed Thrombolysis and Catheter-Directed Thrombolysis Alone for Treatment of Proximal Deep Vein Thrombosis. Cardiovasc Intervent Radiol. 2016;39(8):1115–21.
51. Villalba L, Niknam F. Incidence of residual deep incompetence post successful an-giojet thrombectomy of extensive ilio-femoral DVT. Int Angiol. 2018;37:58.
52. Vogel D, Walsh E, Chen JT, Comerota AJ. Mode of thrombolytic therapy and residual obstruction do not affect valve function. J Vasc Surg. 2011;53(1):261.
53. Wagenhäuser MU, Dimopoulos C, Antakyali K, Meyer-Janiszewski YK, Mulorz J, Ibing W, et al. Clinical outcomes after direct and indirect surgical venous thrombectomy for inferior vena cava thrombosis. J Vasc Surg Venous Lymphatic Disord. 2019;7(3):333-343.e2.
54. Wang CN, Deng HR. Percutaneous Endovenous Intervention Plus Anticoagulation versus Anticoagulation Alone for Treating Patients with Proximal Deep Vein Thrombosis: A Meta-analysis and Systematic Review. Ann Vasc Surg. 2018;49:39–48.
55. Wang H, Gao Y. Efficacy and safety of catheter-directed thrombolysis and anticoagulation for deep vein thrombosis: A meta analysis. Chin J Evid-Based Med. 2018;18(9):953–7.
56. Weber J, Fagan N, Patel M. Lower extremity deep venous thrombosis catheter-directed thrombolysis in pediatric patients: Single-center experience. Pediatr Radiol. 2021;51:S174.
57. Wong PC, Chan YC, Law Y, Cheng SW. Systematic review of percutaneous mechanical thrombectomy in the treatment of acute iliofemoral deep vein thrombosis. Surg Pract. 2017;21:12–3.
58. Xue G, Ni Q, Yang S, Chen J, Ye M, Zhang L. Percutaneous mechanical thrombectomy in the treatment of acute deep venous thrombosis. Int Angiol. 2018;37:11.
59. Yu Z, Lin Z, Lang D. Long-term efficacy of percutaneous mechanical thrombectomy combined with stent implantation in treatment of acute iliofemoral venous thrombosis. Zhejiang Da Xue Xue Bao Yi Xue Ban. 2018;47(6):623–7.
60. Yue TH, Huang J, Zhao H, Zhao SM, Men ZJ. Two therapeutic approaches for acute iliofemoral venous thrombosis: A comparative study. J Intervent Radiol. 2012;21(3):198–201.
61. Zeinali AMH, Jenab Y, Ariannejad H, Kassaian SE, Alidoosti M, Aghajani H, et al. Catheter-directed thrombolysis in acute iliofemoral deep vein thrombosis with or without stenting: A case series. J Tehran Uni Heart Cent. 2018;13(4):186–90.
62. Zhang X, Ren Q, Jiang X, Sun J, Gong J, Tang B, et al. A prospective randomized trial of catheter-directed thrombolysis with additional balloon dilatation for iliofemoral deep venous thrombosis: A single-center experience. Cardiovasc Intervent Radiol. 2014;37(4):958–68.
63. Zhao H, Hu H, Rao M, Wu Z, Ren Q, Wu J, et al. Percutaneous mechanical thrombectomy for treatment of acute iliofemoral vein thrombi. 经皮机械性血栓清除术治疗急性髂股静脉血栓. 2020;17(7):393–7.
64. Ashrafi M, Ahmad SB, Antoniou SA, Khan T, Antoniou GA. Treatment Strategies for Proximal Deep Vein Thrombosis: A Network Meta-analysis of Randomised Controlled Trials. Eur J Vasc Endovasc Surg. 2022;63(2):323–34.
65. Qian C, Chen GP, Lou WS, Wang T, Li YH. The perplexity of catheter-directed thrombolysis for deep venous thrombosis: the approaches play an important role. J Thromb Thrombolysis. 2021;51(3):757–66.
66. Cao W, Shi H, Lu W, Chen Q. Mid- and Short-Term Efficacy of Percutaneous Mechanical Thrombectomy in the Treatment of Acute Iliofemoral Deep Vein Thrombosis. Ann Vasc Surg. 2020;68:179–84.
67. Xu YD, Zhong BY, Yang C, Cai XS, Hu B, Wang XY, et al. Comparison of catheter-directed thrombolysis with and without percutaneous mechanical thrombectomy for subacute iliofemoral deep vein thrombosis. PHLEBOLOGY. 2020;35(8):589–96.
68. Jiang C, Zhao Y, Wang X, Liu H, Tan TW, Li F. Midterm outcome of pharmacomechanical catheter-directed thrombolysis combined with stenting for treatment of iliac vein compression syndrome with acute iliofemoral deep venous thrombosis. J Vasc Surg Venous Lymphat Disord. 2020;8(1):24–30.
69. Rabuffi P, Vagnarelli S, Bruni A, Gallucci M, Ambrogi C, Passaro G, et al. Pharmacomechanical catheter-directed thrombolysis for acute iliofemoral deep vein thrombosis: our case series. Eur Rev Med Pharmacol Sci. 2019;23(5):2244–52.
70. Tang T, Chen L, Chen J, Mei T, Lu Y. Pharmacomechanical Thrombectomy Versus Catheter-Directed Thrombolysis for Iliofemoral Deep Vein Thrombosis: A Meta-Analysis of Clinical Trials. Clin Appl Thromb Hemost. 2019;25:1076029618821190.
71. Taha MA, Busuttil A, Bootun R, Davies AH. A systematic review on the use of deep venous stenting for acute venous thrombosis of the lower limb. PHLEBOLOGY. 2019;34(2):115–27.
72. Murphy EH, Davis CM, Journeycake JM, DeMuth RP, Arko FR. Symptomatic ileofemoral DVT after onset of oral contraceptive use in women with previously undiagnosed May-Thurner Syndrome. J Vasc Surg. 2009;49(3):697–703.
73. Ockert S, von Allmen M, Heidemann M, Brusa J, Duwe J, Seelos R. Acute Venous Iliofemoral Thrombosis: Early Surgical Thrombectomy Is Effective and Durable. Ann Vasc Surg. 2018;46:314–21.
74. Liu G, Qin J, Cui C, Ye K, Shi H, Liu X, et al. Comparison of Direct Iliofemoral Stenting Following AngioJet Rheolytic Thrombectomy vs Staged Stenting After AngioJet Rheolytic Thrombectomy Plus Catheter-Directed Thrombolysis in Patients With Acute Deep Vein Thrombosis. J Endovasc Ther. 2018;25(1):133–9.
75. Rodriguez LE, Aboukheir-Aboukheir A, Figueroa-Vicente R, Soler-Bernardini H, Bolanos-Avila G, Torruella-Bartolomei LJ, et al. Hybrid operative thrombectomy is noninferior to percutaneous techniques for the treatment of acute iliofemoral deep venous thrombosis. J Vasc Surg Venous Lymphat Disord. 2017;5(2):177–84.
76. Kuo TT, Huang CY, Hsu CP, Lee CY. Catheter-directed thrombolysis and pharmacomechanical thrombectomy improve midterm outcome in acute iliofemoral deep vein thrombosis. J Chin Med Assoc. 2017;80(2):72–9.
77. Robertson L, McBride O, Burdess A. Pharmacomechanical thrombectomy for iliofemoral deep vein thrombosis. Cochrane Database Syst Rev. 2016;11(100909747):CD011536.
78. Avgerinos ED, Hager ES, Naddaf A, Dillavou E, Singh M, Chaer RA. Outcomes and predictors of failure of thrombolysis for iliofemoral deep venous thrombosis. J Vasc Surg Venous Lymphat Disord. 2015;3(1):35–41.
79. Bloom AI, Farkas A, Kalish Y, Elchalal U, Spectre G. Pharmacomechanical catheter-directed thrombolysis for pregnancy-related iliofemoral deep vein thrombosis. J Vasc Interv Radiol. 2015;26(7):992–1000.
80. Cakir V, Gulcu A, Akay E, Capar AE, Gencpinar T, Kucuk B, et al. Use of percutaneous aspiration thrombectomy vs. anticoagulation therapy to treat acute iliofemoral venous thrombosis: 1-year follow-up results of a randomised, clinical trial. Cardiovasc Intervent Radiol. 2014;37(4):969–76.
81. Dumantepe M, Tarhan IA, Ozler A. Treatment of chronic deep vein thrombosis using ultrasound accelerated catheter-directed thrombolysis. Eur J Vasc Endovasc Surg. 2013;46(3):366–71.
82. Meissner MH, Gloviczki P, Comerota AJ, Dalsing MC, Eklof BG, Gillespie DL, et al. Early thrombus removal strategies for acute deep venous thrombosis: clinical practice guidelines of the Society for Vascular Surgery and the American Venous Forum. J Vasc Surg. 2012;55(5):1449–62.
83. Casey ET, Murad MH, Zumaeta-Garcia M, Elamin MB, Shi Q, Erwin PJ, et al. Treatment of acute iliofemoral deep vein thrombosis. J Vasc Surg. 2012;55(5):1463–73.
84. Comerota AJ, Grewal N, Martinez JT, Chen JT, Disalle R, Andrews L, et al. Postthrombotic morbidity correlates with residual thrombus following catheter-directed thrombolysis for iliofemoral deep vein thrombosis. J Vasc Surg. 2012;55(3):768–73.
85. Kahn SR, Julian JA, Kearon C, Gu CS, Cohen DJ, Magnuson EA, et al. Quality of life after pharmacomechanical catheter-directed thrombolysis for proximal deep venous thrombosis. J Vasc Surg Venous Lymphat Disord. 2020;8(1):8-23.e18.
86. Mühlberger D, Wenkel M, Papapostolou G, Mumme A, Stücker M, Reich-Schupke S, et al. Surgical thrombectomy for iliofemoral deep vein thrombosis: Patient outcomes at 8.5 years. PLoS One. 2020;15(6):e0235003.
87. Diniz J, Coelho A, Mansilha A. Endovascular treatment of iliofemoral deep venous thrombosis: is there enough evidence to support it? A systematic review with meta-analysis. Int Angiol. 2020;39(2):93–104.
88. Warad DM, Rao AN, Bjarnason H, Rodriguez V. Clinical Outcomes of May-Thurner Syndrome in Pediatric Patients: A Single Institutional Experience. TH Open. 2020;4(3):e189–96.
89. Kuetting D, Luetkens J, Wolter K, Faron A, Kania A, Thomas D. Catheter-Directed Thrombectomy for Highly Symptomatic Patients with Iliofemoral Deep Venous Thrombosis not Responsive to Conservative Treatment. Cardiovasc Intervent Radiol. 2020;43(4):556–64.
90. Wong PC, Chan YC, Law Y, Cheng SWK. Percutaneous mechanical thrombectomy in the treatment of acute iliofemoral deep vein thrombosis: a systematic review. Hong Kong Med J. 2019;25(1):48–57.
91. Patra S, Srinivas BC, Nagesh CM, Reddy B, Manjunath CN. Endovascular management of proximal lower limb deep venous thrombosis - A prospective study with six-month follow-up. Phlebology. 2015;30(7):441–8.
92. Pouncey AL, Gwozdz AM, Johnson OW, Silickas J, Saha P, Thulasidasan N, et al. AngioJet Pharmacomechanical Thrombectomy and Catheter Directed Thrombolysis vs. Catheter Directed Thrombolysis Alone for the Treatment of Iliofemoral Deep Vein Thrombosis: A Single Centre Retrospective Cohort Study. Eur J Vasc Endovasc Surg. 2020;60(4):578–85.
93. Garcia MJ, Sterling KM, Kahn SR, Comerota AJ, Jaff MR, Ouriel K, et al. Ultrasound-Accelerated Thrombolysis and Venoplasty for the Treatment of the Postthrombotic Syndrome: Results of the ACCESS PTS Study. J Am Heart Assoc. 2020;9(3):e013398.
94. Gaballah M, Shi J, Kukreja K, Raffini L, Tarango C, Keller M, et al. Endovascular Thrombolysis in the Management of Iliofemoral Thrombosis in Children: A Multi-Institutional Experience. J Vasc Interv Radiol. 2016;27(4):524–30.
95. Gombert A, Gombert R, Barbati ME, Bruners P, Keszei A, Wittens C, et al. Patency rate and quality of life after ultrasound-accelerated catheter-directed thrombolysis for deep vein thrombosis. Phlebology. 2018;33(4):251–60.
96. Eckenrode G, Baltich Nelson B, Belarmino A, Chen SA, Goel S, Meltzer AJ. Meta-analysis and systematic review of interventional therapy versus anticoagulation for isolated femoropopliteal deep venous thrombosis. J Vasc Surg Venous Lymphat Disord. 2019;7(2):272–6.
97. Weissler EH, Cox MW, Commander SJ, Williams ZF. Restoring venous patency with the ClotTriever following deep vein thrombosis. Ann Vasc Surg [Internet]. 2022.
98. Nie M, Fu J, Sun J, Wang H. Percutaneous Mechanical Thrombectomy for Acute Symptomatic Iliofemoral Deep Venous Thrombosis Patients With Recent Aneurysmal Subarachnoid Hemorrhage. J Endovasc Ther. 2022;15266028221079772.
99. Berencsi A, Dósa E, Nemes B, Hüttl K, Legeza P, Oláh Z, et al. [Endovascular treatment of acute iliofemoral deep venous thrombosis - our results with catheter-directed thrombolysis and AngioJet]. Magy Seb. 2017;70(1):24–31.
100. Jeyabalan G, Marone L, Rhee R, Hirsch S, Makaroun MS, Cho J, et al. Inflow thrombosis does not adversely affect thrombolysis outcomes of symptomatic iliofemoral deep vein thrombosis. J Vasc Surg. 2011;54(2):448–53.
101. Thomas M, Hollingsworth A, Mofidi R. Endovascular Management of Acute Lower Limb Deep Vein Thrombosis: A Systematic Review and Meta-analysis. Ann Vasc Surg. 2019;58:363–70.
102. Rabellino M, Moltini P, Di Caro V, Garcia-Monaco R. Symptomatic Iliofemoral and Iliocaval Venous Thrombosis in Patients With Cancer: Endovascular Treatment. Vasc Endovascular Surg. 2018;52(8):602–6.
103. Gagne PJ, Rajasinghe H, Khoury T. ISOL-8: A Multicenter, Retrospective Study of the Effectiveness of the Trellis-8 to Treat Iliofemoral DVT and Prevent Post-thrombotic Syndrome. J Vasc Surg Venous Lymphat Disord. 2013;1(1):108–9.
104. Cosmi B, Stanek A, Kozak M, Wennberg PW, Kolluri R, Righini M, et al. The Post-thrombotic Syndrome-Prevention and Treatment: VAS-European Independent Foundation in Angiology/Vascular Medicine Position Paper. Front Cardiovasc Med. 2022;9:762443.
105. Jayaraj A, Lucas M, Fuller R, Powell T, Kuykendall R. Improvement following restoration of inline flow argues against comprehensive thrombus removal strategies and for selective stenting in acute symptomatic iliofemoral venous thrombosis. J Vasc Surg Venous Lymphat Disord [Internet]. 2022.
106. Delomez M, Beregi JP, Willoteaux S, Bauchart JJ, Janne d’Othée B, Asseman P, et al. Mechanical thrombectomy in patients with deep venous thrombosis. Cardiovasc Intervent Radiol. 2001;24(1):42–8.
107. Vedantham S, Vesely TM, Sicard GA, Brown D, Rubin B, Sanchez LA, et al. Pharmacomechanical thrombolysis and early stent placement for iliofemoral deep vein thrombosis. J Vasc Interv Radiol. 2004 Jun;15(6):565–74.
108. Kamphausen M, Barbera L, Mumme A, Marpe B, Grossefeld M, Asciutto G, et al. [Clinical and functional results after transfemoral thrombectomy for iliofemoral deep venous thrombosis: a 5-year-follow-up]. Zentralbl Chir. 2005 Oct;130(5):454–61; discussion 461-462.
109. Lee KH, Han H, Lee KJ, Yoon CS, Kim SH, Won JY, et al. Mechanical thrombectomy of acute iliofemoral deep vein thrombosis with use of an Arrow-Trerotola percutaneous thrombectomy device. J Vasc Interv Radiol. 2006 Mar;17(3):487–95.
110. Arko FR, Davis CM, Murphy EH, Smith ST, Timaran CH, Modrall JG, et al. Aggressive percutaneous mechanical thrombectomy of deep venous thrombosis: early clinical results. Arch Surg. 2007 Jun;142(6):513–8; discussion 518-519.

***AIM 3***

1. Ragg J. Artificial Intelligence in the Research of Origins of Vein Insufficiency. J Vasc Surg Venous Lymphatic Disord. 2022;10(2):543–4.
2. Kaul N, Huang HYS. Constitutive modeling of jugular vein-derived venous valve leaflet tissues. J Mech Behav Biomed Mater. 2017;75:50–7.
3. Ragg C. EPS: A New Etiology-Based Classification of Vein Insufficiency. J Vasc Surg Venous Lymphatic Disord. 2020;8(2):318.
4. De Souza G, Pereira AH, Costa LFM, Silva JCCB, Burihan E. Hemodynamic results of femoral vein valve repair. Cardiovasc Surg. 2001;9(2):127–32.
5. Chen HY, Diaz JA, Lurie F, Chambers SD, Kassab GS. Hemodynamics of venous valve pairing and implications on helical flow. J Vasc Surg Venous Lymphatic Disord. 2018;6(4):517-522.e1.
6. Jones C, Hinds M, Pavcnik D. Improved bioprosthetic valve for the treatment of chronic deep venous insufficiency using an autologous endothelial layer. J Vasc Intervent Radiol. 2012;23(3):S7.
7. Jonas K, Marko S, Ronald S, Armin S, Horst C, Heike K, et al. Learning from vessel and valve morphology of the human femoral vein-morphometric and histostructural requirements for artificial valve implants. Surg Radiol Anat. 2018;40(1):S22.
8. Lu W, Pavcnik D, Li YH. Percutaneous placed bioprosthetic venous vaive in the treatment of deep vein reflux: Animal experiments and clinical trials. Chin J Radiol (Zhonghua Fangshexue Zazhi). 2008;42(2):184–8.
9. Gale SS, Shuman S, Beebe HG, Pigott JP, Comerota AJ. Percutaneous venous valve bioprosthesis: initial observations. Vasc Endovascular Surg. 2004;38(3):221–4.
10. Pavcnik D, Uchida B, Kaufman J, Keller FS, Rösch J. Percutaneous venous valve implantation in management of chronic deep venous insufficiency: An overview of our experimental work and early clinical experience. Ceska Radiol. 2007;61(2):129–37.
11. Ma T, Fu W, Ma J. Popliteal vein external banding at the valve-free segment to treat severe chronic venous insufficiency. J Vasc Surg. 2016;64(2):438-445.e1.
12. Teebken OE, Puschmann C, Breitenbach I, Rohde B, Burgwitz K, Haverich A. Preclinical development of tissue-engineered vein valves and venous substitutes using re-endothelialised human vein matrix. Eur J Vasc Endovasc Surg. 2009;37(1):92–102.
13. Ragg C. PVP is Effective and Safe to Restore Valve Function, Best Suitable for Early Stages of Pressure-induced Valve Decompensation. J Vasc Surg Venous Lymphatic Disord. 2021;9(2):551.
14. Jones CM, Hinds MT, Pavcnik D. Retention of an autologous endothelial layer on a bioprosthetic valve for the treatment of chronic deep venous insufficiency. J Vasc Intervent Radiol. 2012;23(5):697–703.
15. Ulloa J, Glickman M, Cifuentes S, Figueroa V. Three-year Results of the Surgical Implantation of the Bioprosthetic Valve: VenoValve, for Patients with Severe Chronic Venous Insufficiency, C5-C6 Disease. J Vasc Surg Venous Lymphatic Disord. 2022;10(2):572.
16. Mogaldea A, Goecke T, Theodoridis K, Haverich A, Cebotari S, Hilfiker A. Tissue Engineering of Vein Valves Based on Decellularized Natural Matrices. Cells Tissues Organs. 2017;204(3):199–209.
17. Syedain ZH, Jenson AC, Patel PS, Feagler C, Bahmer L, Faizer R, et al. Tissue-engineered transcatheter vein valve. Biomaterials [Internet]. 2019;216.
18. Tripathi R, Ktenidis KD. Trapdoor internal valvuloplasty - A new technique for primary deep vein valvular incompetence. Eur J Vasc Endovasc Surg. 2001;22(1):86–9.
19. Ragg C. Vein valve defects and insufficiency in children. Phlebology. 2017;32(2):15.
20. Ulloa JH, Glickman M. One-Year First-in-Human Success for VenoValve in Treating Patients With Severe Deep Venous Insufficiency. Vasc Endovascular Surg. 2022 Apr;56(3):277–83.
21. Ulloa JH, Cifuentes S, Figueroa V, Glickman M. Two-Year Results of a First-In-Human Study in Patients Surgically Implanted With a Bioprosthetic Venous Valve, the VenoValve in Patients With Severe Chronic Venous Insufficiency. Vasc Endovascular Surg. 2023 February 6;15385744231155328.

***AIM 4***

1. Rabinovich A, Cohen J, Cushman M, Houweling A, Shapiro S, Wells P, et al. Inflammation markers and the risk of post thrombotic syndrome: res WU. Blood [Internet]. 2013;122(21).
2. Rabinovich A, Cohen JM, Prandoni P, Kahn SR. Association between thrombophilia and the post-thrombotic syndrome: a systematic review and meta-analysis. Journal of Thrombosis & Haemostasis. 2014;12(1):14–23.
3. Spiezia L, Campello E, Valle FD, Simion C, Colpo A, Simioni P. ABO blood group and the risk of post-thrombotic syndrome. Annals of Hematology. 2018;97(6):1057–60.
4. Franciscis S, Gallelli L, Amato B, Butrico L, Rossi A, Buffone G, et al. Plasma MMP and TIMP evaluation in patients with deep venous thrombosis: could they have a predictive role in the development of post-thrombotic syndrome? INT WOUND J. 2016;13(6):1237–45.
5. Sachdev U, Vodovotz L, Barclay D, Lin Y, Zamora R, Bitner J, et al. Infrared Thermography Confirms Inflammatory Differences in Refluxing Versus Competent Lower Extremity Veins. J Vasc Surg Venous Lymphatic Disord. 2020;8(2):310–1.
6. Bouman AC, Atalay S, Ten Cate H, Ten Wolde M, Ten Cate-Hoek AJ. Biomarkers for post-thrombotic syndrome. J Vasc Surg Venous Lymphat Disord. 2014;2(1):79-88.e3.
7. Gemmati D, Federici F, Catozzi L, Gianesini S, Tacconi G, Scapoli GL, et al. DNA-array of gene variants in venous leg ulcers: detection of prognostic indicators. J Vasc Surg. 2009;50(6):1444–51.
8. Rabinovich A, Cohen JM, Cushman M, Wells PS, Rodger MA, Kovacs MJ, et al. Inflammation markers and their trajectories after deep vein thrombosis in relation to risk of post-thrombotic syndrome. Journal of Thrombosis and Haemostasis. 2015;13(3):398–408.
9. Latella J, Desmarais S, Miron MJ, Roussin A, Joyal F, KASSIS J, et al. Relation between D-dimer level, venous valvular reflux and the development of post-thrombotic syndrome after deep vein thrombosis. Journal of Thrombosis and Haemostasis. 2010;8(10):2169–75.
10. Bouman AC, Smits JJM, Ten Cate H, Ten Cate-Hoek AJ. Markers of coagulation, fibrinolysis and inflammation in relation to post-thrombotic syndrome. Journal of Thrombosis and Haemostasis. 2012;10(8):1532–8.
11. Jünger M, Steins A, Hahn M, Häfner HM. Microcirculatory dysfunction in chronic venous insufficiency (CVI). Microcirculation (New York, NY : 1994). 2000;7(6):S3-12.
12. Ortega MA, Asúnsolo Á, Leal J, Romero B, Alvarez-Rocha MJ, Sainz F, et al. Implication of the PI3K/Akt/mTOR Pathway in the Process of Incompetent Valves in Patients with Chronic Venous Insufficiency and the Relationship with Aging. Oxidative Medicine and Cellular Longevity. 2018;2018:1–14.
13. Ortega MA, Fraile-Martínez O, García-Montero C, Ruiz-Grande F, Álvarez-Mon MA, Monserrat J, et al. Contribution of the Elastic Component and Venous Wall Arterialization in Patients with Venous Reflux. Journal of Personalized Medicine. 2022;12(2):260–260.
14. Bittar LF, Silva LQ da, Orsi FL de A, Zapponi KCS, Mazetto B de M, Paula EV de, et al. Increased inflammation and endothelial markers in patients with late severe post-thrombotic syndrome. PLOS ONE. 2020;15(1):e0227150–e0227150.
15. Rabinovich A, Cohen JM, Kahn SR. Predictive value of markers of inflammation in the postthrombotic syndrome: a systematic review. Thrombosis Research. 2015;136(2):289–97.
16. Bittar LF, Paula EV de, Montalvão SAL, Mello TBT, Annichino-Bizzacchi JM. Severe Post-thrombotic Syndrome is Associated With Higher Levels of Factor VIII. Clinical and Applied Thrombosis/Hemostasis. 2013;19(5):570–3.
17. Sartori M, Favaretto E, Cini M, Legnani C, Palareti G, Cosmi B. D-dimer, FVIII and thrombotic burden in the acute phase of deep vein thrombosis in relation to the risk of post-thrombotic syndrome. Thrombosis Research. 2014;134(2):320–5.
18. Betensky M, Amankwah EK, Brandal S, Everett AD, Goldenberg NA. Plasma fibrinolysis, inflammatory markers, and postthrombotic syndrome: preliminary findings from the Kids-DOTT Biobank. Blood Advances. 2021;5(1):233–9.
19. Smith RK, Golledge J. A systematic review of circulating markers in primary chronic venous insufficiency. Phlebology: The Journal of Venous Disease. 2014;29(9):570–9.
20. Mosmiller LT, Steele KN, Shrader CD, Petrone AB. Evaluation of inflammatory cell biomarkers in chronic venous insufficiency. Phlebology: The Journal of Venous Disease. 2017;32(9):634–40.
21. Karahan O, Yavuz C, Kankilic N, Demirtas S, Tezcan O, Caliskan A, et al. Simple blood tests as predictive markers of disease severity and clinical condition in patients with venous insufficiency. Blood Coagulation & Fibrinolysis. 2016;27(6):684–90.
22. Sachdev U, Vodovotz L, Bitner J, Barclay D, Zamora R, Yin J, et al. Suppressed networks of inflammatory mediators characterize chronic venous insufficiency. Journal of Vascular Surgery: Venous and Lymphatic Disorders. 2018;6(3):358–66.
23. Grudzińska E, Grzegorczyn S, Czuba ZP. Chemokines and Growth Factors Produced by Lymphocytes in the Incompetent Great Saphenous Vein. Mediators of Inflammation. 2019;2019:1–10.
24. Pappas PJ, Teehan EP, Fallek SR, Garcia A, Araki CT, Back TL, et al. Diminished mononuclear cell function is associated with chronic venous insufficiency. Journal of Vascular Surgery. 1995;22(5):580–6.
25. Serralheiro P, Novais A, Cairrão E, Maia C, Costa Almeida C, Verde I. Variability of MMP/TIMP and TGF-β1 Receptors throughout the Clinical Progression of Chronic Venous Disease. International Journal of Molecular Sciences. 2017;19(1):6–6.
26. Pappas PJ, You R, Rameshwar P, Gorti R, DeFouw DO, Phillips CK, et al. Dermal tissue fibrosis in patients with chronic venous insufficiency is associated with increased transforming growth factor-β1 gene expression and protein production. Journal of Vascular Surgery. 1999;30(6):1129–45.
27. Tiwary SK, Kumar A, Mishra SP, Kumar P, Khanna AK. Study of association of varicose veins and inflammation by inflammatory markers. Phlebology: The Journal of Venous Disease. 2020;35(9):679–85.
28. Lattimer CR, Kalodiki E, Geroulakos G, Hoppensteadt D, Fareed J. Are Inflammatory Biomarkers Increased in Varicose Vein Blood? Clinical and Applied Thrombosis/Hemostasis. 2016;22(7):656–64.
29. Lattimer CR, Kalodiki E, Geroulakos G, Syed D, Hoppensteadt D, Fareed J. D-Dimer Levels are Significantly Increased in Blood Taken From Varicose Veins Compared With Antecubital Blood From the Same Patient. Angiology. 2015;66(9):882–8.
30. Anwar MA, Vorkas P, Li J, Shalhoub J, Lim CS, Want E, et al. Differential Metabolic Phenotype of Human Varicose Veins Tissue and Their Utility in Understanding Disease Pathogenesis and Identifying Potential Prognostic Biomarkers. Journal of Vascular Surgery: Venous and Lymphatic Disorders. 2014;2(1):113–113.
31. Yasim A, Kilinç M, Aral M, Oksuz H, Kabalci M, Eroglu E, et al. Serum concentration of procoagulant, endothelial and oxidative stress markers in early primary varicose veins. Phlebology: The Journal of Venous Disease. 2008;23(1):15–20.
32. Gemmati D, Tognazzo S, Serino ML, Fogato L, Carandina S, De Palma M, et al. Factor XIII V34L polymorphism modulates the risk of chronic venous leg ulcer progression and extension. Wound Repair and Regeneration. 2004;12(5):512–7.
33. Bouman AC, Cheung YW, Spronk HM, Schalkwijk CG, Ten Cate H, Ten Wolde M, et al. Biomarkers for post thrombotic syndrome: A case-control study. Thrombosis Research. 2014;134(2):369–75.
34. Saito S, Trovato MJ, You R, Lal BK, Fasehun F, Padberg FT, et al. Role of matrix metalloproteinases 1, 2, and 9 and tissue inhibitor of matrix metalloproteinase-1 in chronic venous insufficiency. Journal of Vascular Surgery. 2001;34(5):930–8.
35. Bharath V, Kahn SR, Lazo-Langner A. Genetic polymorphisms of vein wall remodeling in chronic venous disease: A narrative and systematic review. Blood. 2014;124(8):1242–50.
36. Onida S, Tan MKH, Kafeza M, Bergner RT, Shalhoub J, Holmes E, et al. Metabolic Phenotyping in Venous Disease: The Need for Standardization. Journal of Proteome Research. 2019;18(11):3809–20.
37. He R, Cai H, Jiang Y, Liu R, Zhou Y, Qin Y, et al. Integrative analysis prioritizes the relevant genes and risk factors for chronic venous disease. Journal of Vascular Surgery: Venous and Lymphatic Disorders. 2022;10(3):738-748.e5.
